# Supplementary figures and images for: Neural variability reliably encodes interindividual differences in the perception of pain intensity
Source: PLoS Biol. 2025 Oct 27;23(10):e3003470. doi: 10.1371/journal.pbio.3003470 (PMC12574952; doi:10.1371/journal.pbio.3003470)

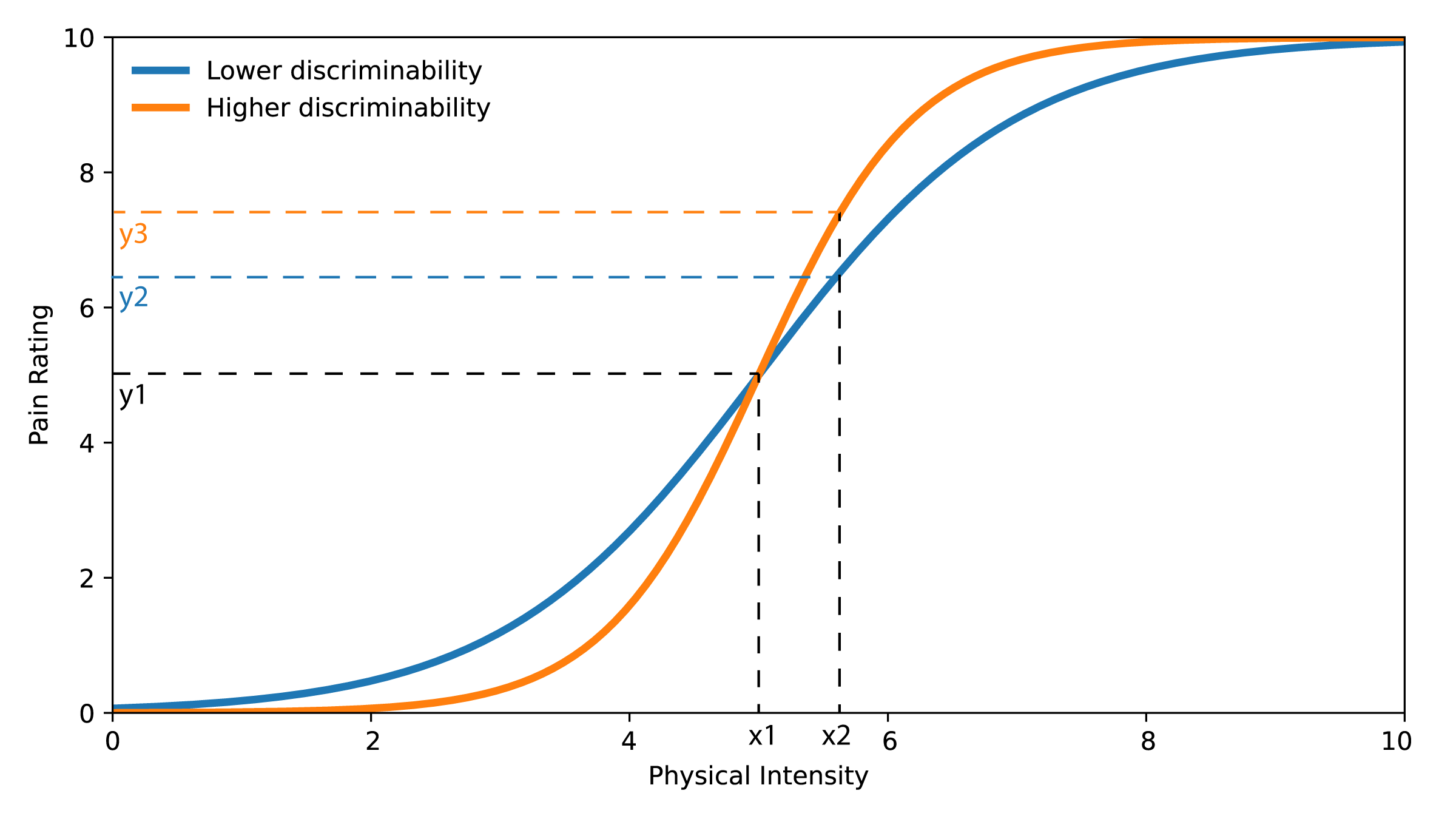

Supplement: S1 Fig — Given two fixed and medium physical intensities x1 and x2, the rating differences y3 − y1 and y2 − y1 can be viewed as the slope of psychophysical functions. Individuals with higher discriminability have a larger slope (i.e., y3 − y1 > y2 − y1). (TIF) [file pbio.3003470.s001.tif]

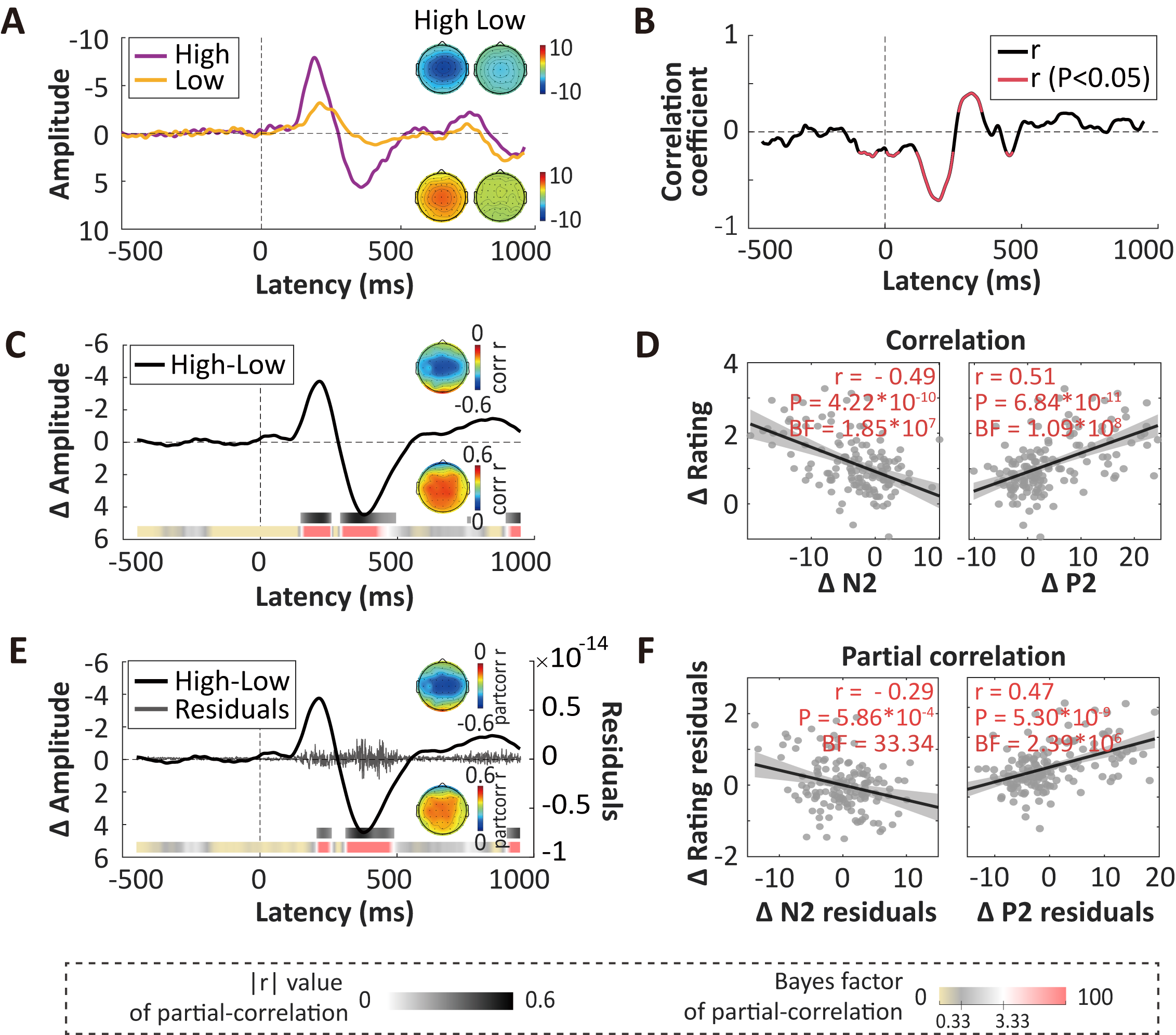

Supplement: S2 Fig — (A) Comparison of ERP amplitudes derived from 100 ms sliding windows between conditions of high- and low-intensity stimuli in Dataset 1. (B) Correlation between amplitude difference and SD difference. Black segments of the curve illustrate time points where correlations were not significant after FDR correction, while red segments illustrate time points with significant correlations. (C, D) Correlations between ERP amplitude difference and pain intensity discriminability. ERP amplitude differences around N2 and P2 peaks were significantly correlated with pain intensity discriminability. (E, F) Partial correlations between ERP amplitude difference and pain intensity discriminability controlling for SD. The light-colored “bursty” curve in panel E represents the subject-averaged residuals of ∆amplitude after regressing out ∆SD. Partial correlations were calculated while controlling for ∆amplitude. Note that the gray bars represent Pearson’s r values at time points where significant correlations were observed after FDR correction. The color bars underneath display the corresponding BF values for the correlations. Part-corr is short for partial correlation. BF is short for Bayes factor. Error bars are 95% confidence intervals. The data underlying this Figure can be found in https://doi.org/10.17605/OSF.IO/QTV8A. (TIF) [file pbio.3003470.s002.tif]

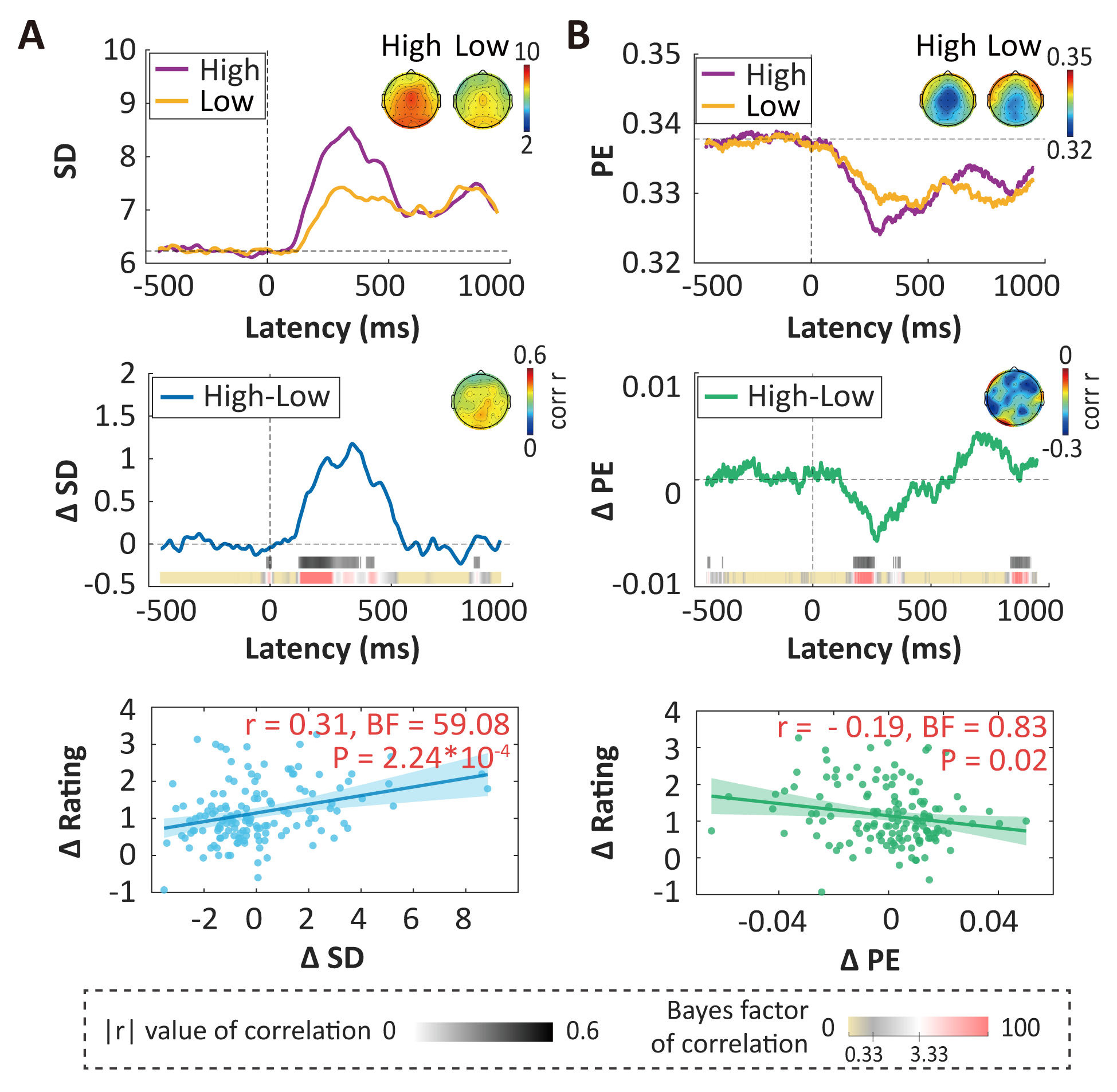

Supplement: S4 Fig — (A) Temporal SD of induced EEG responses and correlations between ∆SD and pain intensity discriminability in Dataset 1. (B) Temporal PE of induced EEG responses and correlations between ∆PE and pain intensity discriminability in Dataset 1. Note that the gray bars represent Pearson’s r values at time points where significant correlations were observed after FDR correction. The color bars underneath display the corresponding Bayes factor values for the correlations. Error bars are 95% confidence intervals. The data underlying this Figure can be found in https://doi.org/10.17605/OSF.IO/QTV8A. (TIF) [file pbio.3003470.s004.tif]

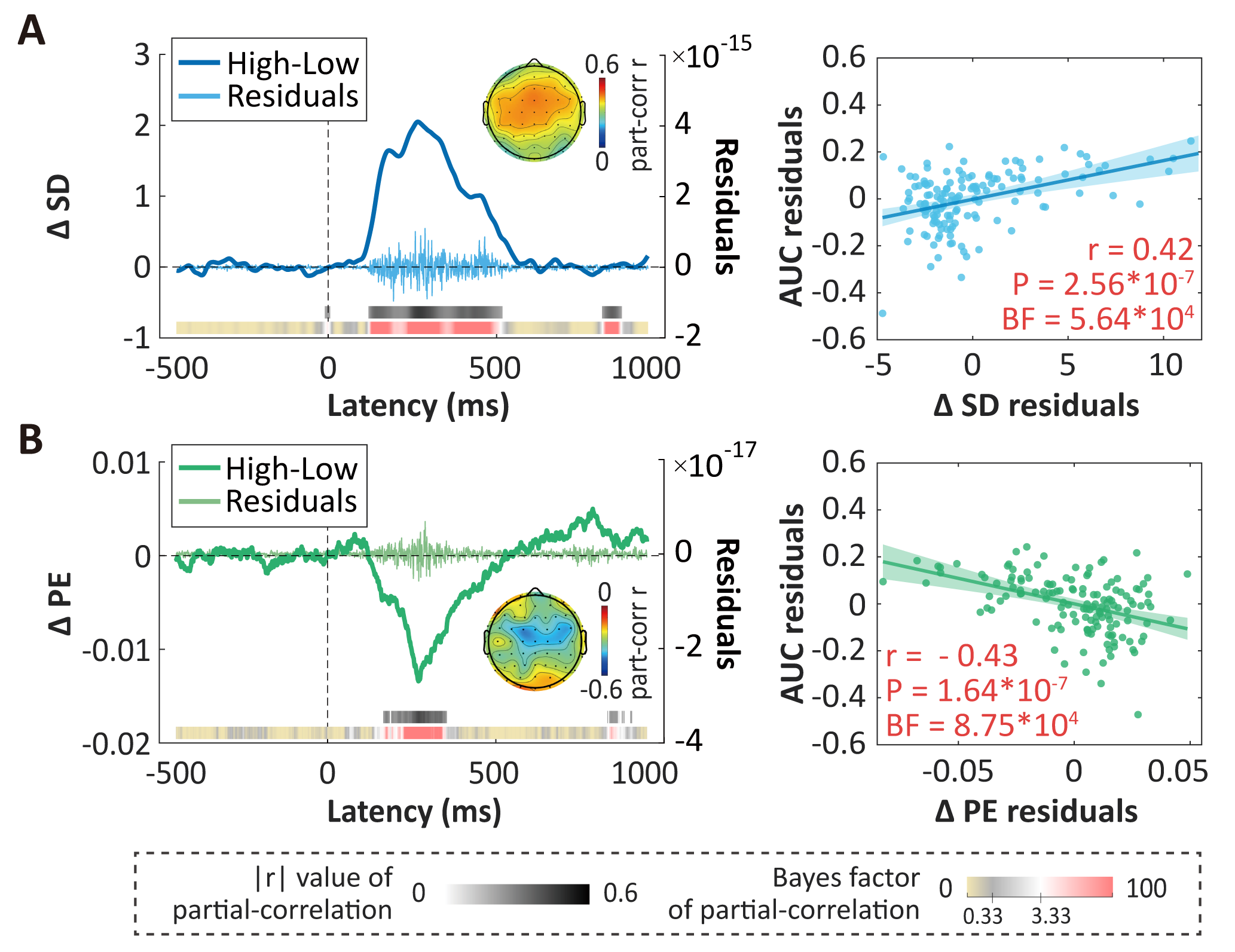

Supplement: S5 Fig — (A) Neural variability measured by temporal SD and its partial correlation with pain intensity discriminability measured by AUC controlling for the amplitude of ERPs. (B) Neural variability measured by temporal PE and its partial correlation with AUC controlling for the amplitude of ERPs. Note that the gray bars represent Pearson’s r values at time points where significant correlations were observed after FDR correction. The color bars underneath display the corresponding Bayes factor values for the correlations. Part-corr is short for partial correlation. The light-colored “bursty” curves represent the subject-averaged residuals of SD/PE after regressing out ERP amplitude. The significance of both indices demonstrates the stability and robustness of neural variability as an indicator of pain intensity discriminability. In scatterplots, r represents r value of partial correlation and error bars are 95% confidence intervals. The data underlying this Figure can be found in https://doi.org/10.17605/OSF.IO/QTV8A. (TIF) [file pbio.3003470.s005.tif]

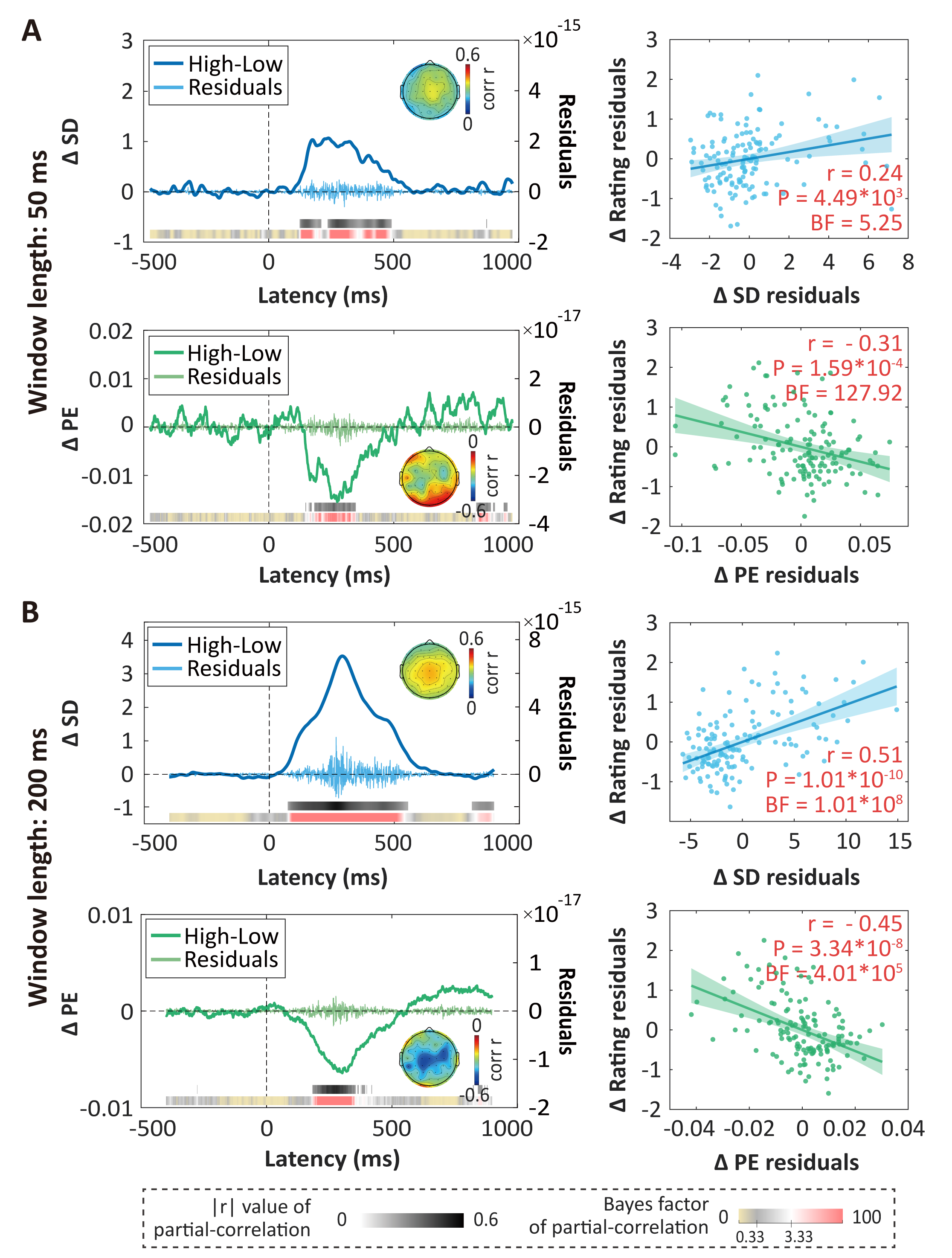

Supplement: S6 Fig — (A, B) Partial correlations of neural variability and pain intensity discriminability controlling for ERP amplitude. The neural variability was calculated with sliding windows of 50 ms in (A) and 200 ms in (B). Consistent significant findings existed in both window sizes. Note that the gray bars represent Pearson’s r values at time points where significant correlations were observed after FDR correction. The color bars underneath display the corresponding Bayes factor values for the correlations. The light-colored “bursty” curves represent the subject-averaged residuals of SD/PE after regressing out ERP amplitude. In scatterplots, r represents r value of partial correlation and error bars are 95% confidence intervals. The data underlying this Figure can be found in https://doi.org/10.17605/OSF.IO/QTV8A. (TIF) [file pbio.3003470.s006.tif]

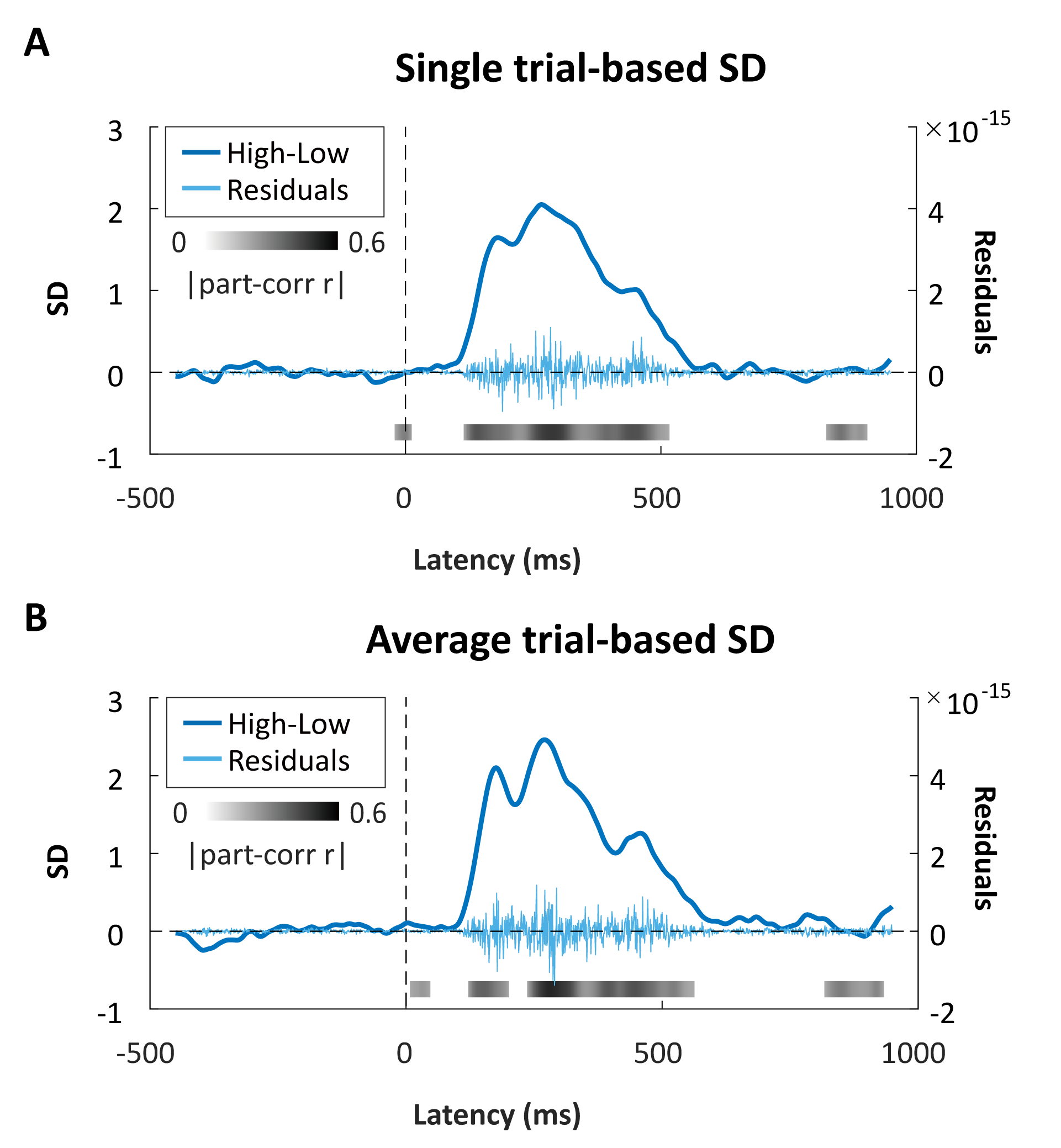

Supplement: S7 Fig — (A, B) Neural variability based on single-trial SD (A) and average trial SD (B) showed similar partial correlations with pain intensity discriminability after regressing out mean ERP amplitude. Note that the gray bars represent Pearson’s r values at time points where significant correlations were observed after FDR correction. Part-corr is short for partial correlation. The data underlying this Figure can be found in https://doi.org/10.17605/OSF.IO/QTV8A. (TIF) [file pbio.3003470.s007.tif]

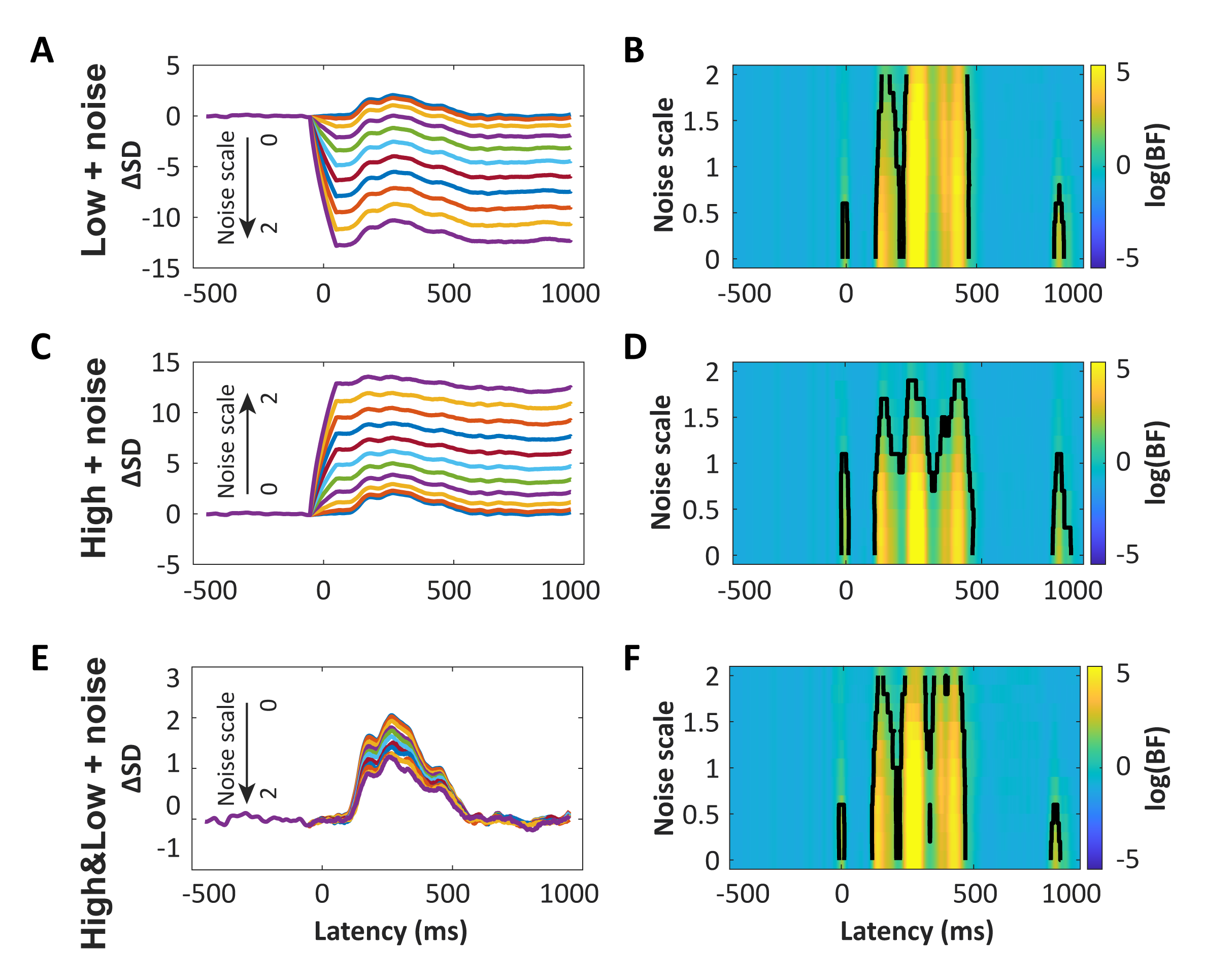

Supplement: S8 Fig — (A) ∆SD time series when different levels of noise were added to the low-intensity condition. As the added noise level increased, SD in the high-intensity condition became smaller than that in the low-intensity condition. Noise scale represents the level of noise added. Eleven noise scales (0–2 in steps of 0.2) were tested. A noise scale of s means s times the baseline noise in single-trial EEG signals was added. (B) Bayes factor (BF) for partial correlations between pain intensity discriminability and neural variability when noise was added to the low-intensity condition while controlling for ERP amplitude differences. Enclosed areas represent correlations with log10(BF) ≥ 0.5, namely, BF ≥ 3.3. Adding noise to the low-intensity condition had no substantial effect on the correlation between pain intensity discriminability and neural variability. (C) ∆SD time series when different levels of noise were added to the high-intensity condition. (D) BF for partial correlations between pain intensity discriminability and neural variability when different levels of noise were added to the high-intensity condition while controlling for ERP amplitude differences. Adding noise to the high-intensity condition also had no substantial effect on the correlation between pain intensity discriminability and neural variability. (E) ∆SD time series when different levels of noise were added to the high- and low-intensity conditions simultaneously. (F) BF for partial correlations between pain intensity discriminability and neural variability when different levels of noise were added to the high- and low-intensity conditions while controlling for ERP amplitude differences. Adding noise also had no substantial effect on the correlation between pain intensity discriminability and neural variability. The data underlying this Figure can be found in https://doi.org/10.17605/OSF.IO/QTV8A. (TIF) [file pbio.3003470.s008.tif]

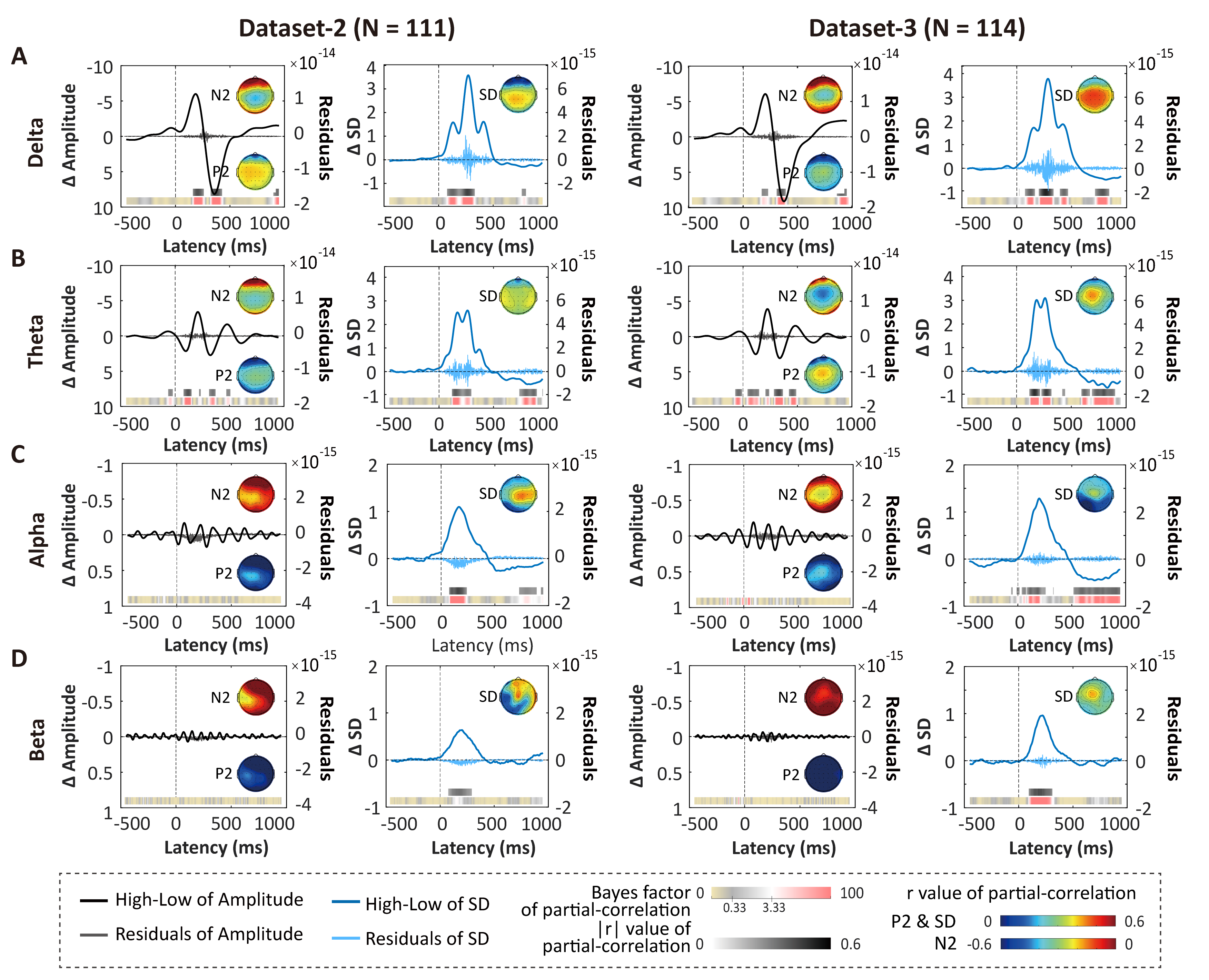

Supplement: S9 Fig — (A–D) Differential amplitude (black curves), neural variability (blue curves) at the delta (1–4 Hz), theta (4–8 Hz), alpha (8–12 Hz), and beta (12–30 Hz) bands, and their partial correlations with pain intensity discriminability while controlling for each other in Datasets 2 and 3. Note that the gray bars represent Pearson’s r values at time points where significant correlations were observed after FDR correction. The color bars underneath display the corresponding Bayes factor values for the correlations. The topographies represent partial r values within a ±10 ms window around the peak. For ∆Amplitude calculations in A and B, the latencies of N2 and P2 peaks were utilized, while for C and D, where clear ERPs were not observed, latencies corresponding to the minimal and maximal values within the 100–500 ms time window were used. The data underlying this Figure can be found in https://doi.org/10.17605/OSF.IO/QTV8A. (TIF) [file pbio.3003470.s009.tif]

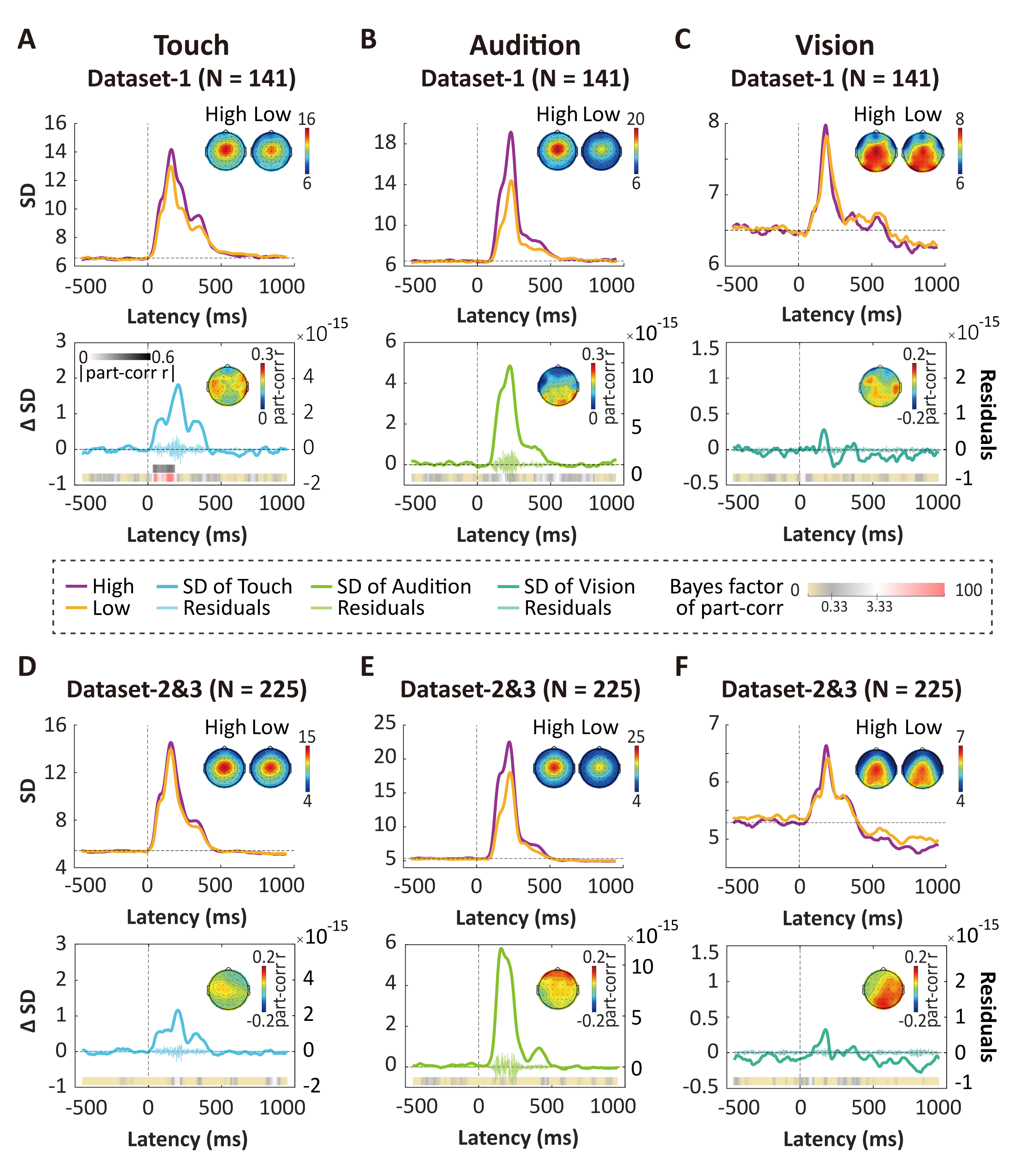

Supplement: S10 Fig — (A–C) Neural variability, and its partial correlations with sensory intensity discriminability of tactile, auditory, and visual stimuli in Dataset 1. Gray bars represent Pearson’s r values at time points where significant correlations were observed (FDR-corrected). The color bars underneath display the corresponding Bayes factor values for the correlations. The light-colored “bursty” curves represent the subject-averaged residuals of ∆SD after regressing out ∆amplitude at each time point. For tactile modality, significant partial correlations while controlling for mean amplitude differences were found at 35–180 ms, but no significance was observed around the peak of neural variability (see Fig 5). No significance was observed for either auditory or visual modalities in partial correlations while controlling for mean amplitude differences of ERPs. (D–F) Neural variabilities and their partial correlations with respective sensory intensity discriminability in Datasets 2&3. No significant partial correlations while controlling for mean amplitude differences were observed for any of these sensory modalities. Part-corr is short for partial correlation. The data underlying this Figure can be found in https://doi.org/10.17605/OSF.IO/QTV8A. (TIF) [file pbio.3003470.s010.tif]

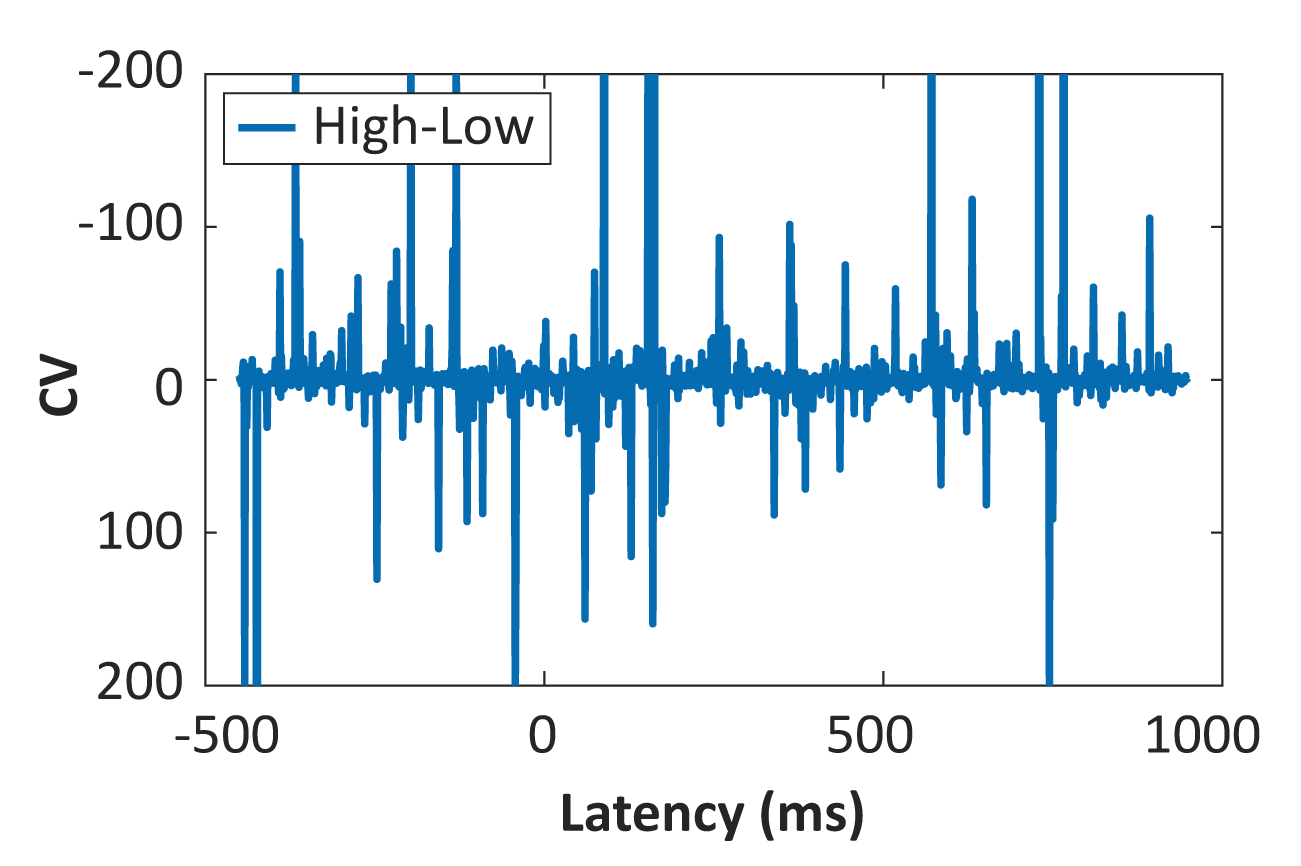

Supplement: S11 Fig — The coefficient of variation (CV) normalizes SD by dividing it by the mean. CV was extremely unstable in many time points. Note that the y axis is capped at ±150 for better visualization. Actually, CV values ranged from a maximum of 2,442 to a minimum of −5,017, illustrating its instability in conditions with low mean signal amplitudes. The data underlying this Figure can be found in https://doi.org/10.17605/OSF.IO/QTV8A. (TIF) [file pbio.3003470.s011.tif]
