# Supplementary material for: Neural variability reliably encodes interindividual differences in the perception of pain intensity
Source: PLoS Biol. 2025 Oct 27;23(10):e3003470. doi: 10.1371/journal.pbio.3003470 (PMC12574952; doi:10.1371/journal.pbio.3003470)
Supplement: Attachment — Submitted filename: Response to Reviewers.docx [file pbio.3003470.s012.docx]

**Reviewer #1:**

The premise and rationale are weak. I am not convinced that the topic of EEG variability is an exact measurable science. Neural activity in the brain by definition is stochastic, and that is a separate topic that requires a different analytical approach.

**Reply**: We thank the Reviewer for the thoughtful comment. It is true that EEG variability has historically received less attention than more established features of EEG signals such as ERPs and neural oscillations. However, there is an increasing number of research investigating the variability of EEG data, including temporal moment-to-moment variability and trial-to-trial variability (Dinstein et al., 2015; Garrett et al., 2013; Waschke et al., 2021). Quantified by metrics such as standard deviation, Fano factor, permutation entropy, and multi-scale entropy (Waschke et al., 2021), EEG variability has been associated with perceptual abilities (Arazi et al., 2017), conscious perception (Schurger et al., 2015), face memory performance (McIntosh et al., 2008), decision bias (Kloosterman et al., 2020), among other cognitive and behavioral outcomes (Waschke et al., 2021). We agree that neural activity is likely stochastic, and this is precisely why investigating variability, as a quantifiable feature, can offer complementary insights into the neural mechanisms underlying cognition and behavior. While a general consensus on the measurability and cognitive/clinical relevance is unlikely at present, we believe EEG variability is a valuable topic to investigate. Continued empirical research is essential to clarify the relationship between EEG variability and cognitive and clinical variables, if there is any.

Nor do I find pain discriminability clinically relevant (evidence presented is not robust).

**Reply**: We thank the Reviewer for raising this important point. We acknowledge that some of the studies we originally cited are relatively dated and based on small samples, largely reflecting the limited number of investigations specifically focused on pain intensity discriminability. However, we believe that this does not inherently undermine their robustness. In fact, more recent research continues to support the clinical relevance of pain intensity discriminability. For example, Hollins et al. (2012) found that patients with sickle cell disease, which is marked by intense pain, had impaired pain intensity discriminability. Some other studies have found that improving somatosensory discrimination through tactile training could reduce cortical reorganization and pain symptoms in chronic pain patients (Flor et al., 2001; Moseley et al., 2008; Pleger et al., 2005). While not directly related to pain intensity discriminability, they suggest a link between discrimination ability and chronic pain. These more recent studies have been cited in support of the clinical relevance of discriminability in the revised manuscript (lines 64~69).

Additionally, in our data, we found that the association between neural variability and pain intensity discriminability was disrupted to some extent on the neuropathic pain-affected side in patients with postherpetic neuralgia. This finding, although preliminary, suggests that changes in discrimination ability may reflect clinically relevant alterations in sensory processing. Nevertheless, we acknowledge that the clinical implications of our findings need further validation in larger and more diverse patient populations. Without broader testing in various chronic pain patients, the clinical relevance of neural variability is still limited. This limitation has been made clear in the revised manuscript (lines 561~565).

There are several approaches to studying pain and EEG that are not ERP based, that were not acknowledged. Several claims were also not well supported or a stretch (for e.g. claims about gamma, etc.)

**Reply**: To avoid potential confusion, we have clarified in the revised manuscript that our study primarily adopts an ERP-based approach. This choice was motivated by both methodological and empirical considerations. First, while alternative EEG approaches such as gamma band oscillations have proven to preferentially encode pain perception, they have lower signal-to-noise ratio than ERP components like N2 and P2 (Bromm & Treede, 1987; Li et al., 2023). Second, recent evidence suggested that event-related spectral perturbation such as alpha oscillations did not show consistent associations with pain intensity discriminability (Zhang et al., 2022), while ERP amplitude has been reliably associated with pain intensity discriminability (Zhang et al., 2022). Given our specific interest in the neural correlates of pain intensity discrimination, we therefore focused on ERPs as the most suitable and empirically supported approach for this investigation. These points have been highlighted in the revised manuscript (lines 96~99).

Methods seem exceptionally obfuscated and almost impossible to follow, especially with figures containing exceedingly small fonts.

**Reply**: We apologize for the lack of clarity in the presentation of our methods and figures. In response to the Reviewer’s comment, we have thoroughly revised the Methods section to enhance readability and improve the logical flow of the procedures. Additionally, we have uploaded high-resolution versions of all figures with larger and more legible fonts. Please note that while the figures may appear compressed in the PDF version due to automatic formatting, the original high-quality versions are available in the submission files.

Datasets were not standardized (different systems, different sample sizes, variable age groups) and different conclusions were drawn from distinct groups.

**Reply**: We thank the Reviewer for this important observation. We acknowledge the substantial heterogeneity across datasets in terms of EEG recording systems, sample sizes, age groups, and so on. While we did not standardize datasets in our analyses, we believe it is a strength rather than a weakness as the heterogeneity ensures our findings’ generalizability. Please note that to avoid potential confounding effects, we did not pool all datasets, but instead analyzed different datasets separately. This approach ensured that each analysis was conducted on a homogeneous dataset. Dataset heterogeneity thus has no effect on most of our analyses. The fact that consistent results from these heterogeneous datasets support the robustness and potential generalizability of our key finding that neural variability encodes pain intensity discriminability. We have highlighted this point in the revised manuscript (lines 594~598)

Cleaning of artifacts, a critical step in EEG analysis, was not robust or up to par with current standards.

**Reply**: To the best of our knowledge, our preprocessing pipeline aligns with other EEG studies (Nickel et al., 2022; Valentini et al., 2023). Nevertheless, we recognize the importance of validating our results using more stringent artifact-cleaning procedures. To address this concern, we have included a new dataset (May et al., 2025) and reanalyzed it with the preprocessing pipeline DISCOVER-EEG developed by the group that publicly shared the dataset (Gil Ávila et al., 2023). This pipeline is more aggressive than ours and includes the following 8 steps: (1) line noise removal with frequency-domain (multi-taper) regression as implemented in the EEGLAB function *pop_cleanline()*; (2) bandpass filtering between 1 and 100 Hz; (3) bad channel rejection with the EEGLAB function *pop_clean_rawdata()*; (4) re-referencing to average; (5) removal of independent components with a ≥ 80% probability of being eye movement and muscle artifacts as identified by the ICLabel classifier (Pion-Tonachini et al., 2019); (6) bad channel interpolation with spherical splines; (7) bad time segments removal with the Artifact Subspace Reconstruction method (Mullen et al., 2015); and (8) epoching from -500 ms to 1000 ms. Default parameters were used for line noise removal, bad channel rejection, ICA, and bad time segments removal. The epoched data were then low-pass filtered at 30 Hz to remove high frequency noise.

This new dataset produced findings that well support our key claim that neural variability correlates with pain intensity discriminability (Figure 7; also see Figure R1 below), further demonstrating the robustness of our original findings. The new preprocessing pipelines and results have been added to our revised manuscript (lines 354~360, and lines 685~701). Moreover, we have shared all data and analysis code on OSF (https://osf.io/qtv8a/) to ensure transparency and reproducibility.

**Figure R1. Neural variability as an indicator of pain intensity discriminability in Datasets 4~5 with multiple levels of nociceptive laser stimuli.**

**(A&C)** Pain intensity ratings (left panel) and temporal SD (right panel) in Dataset 4 (**A**: healthy subjects, N = 96) and Dataset 5 (**C**: healthy subjects, N = 144). Notably, ratings were significantly different across stimulus intensities. **(B&D)** Correlations between ∆SD around the peak and ∆Rating in all pairs of stimulus intensities. For Dataset 4 (**B**), significant correlations were found in discriminating between intensities with a 0.5 J difference (upper panels), as well as between 3.5 J and 2.5 J (middle lower panel). For Dataset 5 **(D)**, significant correlations were found in all pairs of stimulus intensities. BF is short for Bayes Factor. Error bars in the left panels of A and C are standard errors of means, and error bars in B and D represent 95% confidence intervals.

Finally interpretation of results is self-evident and superficial ('one possible explanation is that pain is a warning signal indicating potential harm, therefore pain may exhibit higher alertness and attention than other non pain perception').

These limitations impeded the review and assessment of the scientific merits.

**Reply**: We thank the Reviewer for highlighting the need for a deeper and more theoretically grounded interpretation of our findings. In response, we have revised the discussion to replace the initial superficial explanation with an interpretation grounded in Bayesian inference theory, as suggested by Reviewer 3. In accord with the Bayesian brain hypothesis, pain perception can be theorized as a Bayesian inference process, which posits that perceived pain is the combination of bottom-up nociceptive inputs and top-down pain predictions weighted by their relative precision (Tabor et al., 2017). This framework has received increasing empirical support across pain and other sensory modalities (Coll et al., 2024; de Lange et al., 2018; Mulders et al., 2023; Onysk et al., 2024). From this perspective, neural variability could indeed reflect perceptual uncertainty or shifts in attentional weighting in the prediction of pain. Consequently, differential neural variability in the high and low intensity conditions may signal varying precision of predictions in different conditions, which then results in change in pain intensity discriminability. This interpretation aligns with previous studies linking neural variability to perceptual uncertainty (Echeveste et al., 2020; Orbán et al., 2016) and recent work demonstrating correlations between moment-to-moment variability of BOLD signals and perceptual precision in a learning task (Skowron et al., 2025). Although uncertainty in pain predictions has been associated with nociceptive-evoked ERP responses (Mulders et al., 2023), there remains a lack of pain research directly investigating the relationship between pain prediction precision and neural variability. Future studies are thus in need to directly test the role of pain prediction precision in the association between neural variability and pain intensity discrimination. These points have been discussed in the revised manuscript (lines 491~507).

**Reviewer 2**

General Comments:

The study investigates the relationship between the variability of brain responses (as recorded by EEG) and pain discriminability in both healthy human participants and patients with chronic pain. The topic of the functional role of neural variability is timely and intriguing. The database is impressive, the manuscript is well-written, and the methods appear sound. The results are clearly presented. However, the relevance of the topic, the theoretical framework, and the functional significance of the findings are not entirely clear. The overall relevance of the results can only be fully assessed once these points have been further clarified.

**Reply**: We thank the Reviewer for their positive and constructive feedback. In response to the Reviewer’s concerns, we have provided detailed point-by-point replies below.

Specific Comments:

“1. Definition/Operationalization of Discriminability:

The study addresses discriminability, which can refer to either the characteristics of sensory stimuli (i.e., the quality of being discriminable) or the individual's ability to discriminate. The present study focuses on the latter, operationalizing discriminability as the difference in subjective intensity ratings between two stimuli with objective differences in stimulus intensity. However, it is not clear whether and how this difference in ratings truly reflects discriminability (i.e., the ability to discriminate), or whether it could simply reflect differences in internal scaling of pain intensity and/or individual variations in using the rating scales. Clarifying this point is crucial for properly interpreting the findings. Furthermore, the ability to discriminate is not limited to differentiating between stimulus intensities—it could also refer to the ability to discriminate locations and/or temporal sequences. As the current study only addresses one specific type of discriminability (stimulus intensity), this distinction should be explicitly stated.”

**Reply**: We thank the Reviewer for raising this important point regarding the definition and operationalization of pain discriminability. We agree that internal scaling of pain intensity and/or individual variations in using rating scales may influence our findings. In our study, we operationalized pain discriminability as the difference in subjective pain ratings between stimuli of two different intensities. This approach can control for this internal scaling in some sense, so long as one makes the assumption that the internal rating scale remains relatively consistent across different stimulus intensities for the same individual. However, we acknowledge that raw rating differences do not fully account for potential scaling biases, as such biases may affect not only the mean but also the distribution of ratings. To address this concern, we conducted control analyses using a signal detection theory (SDT)-based, non-parametric measure of discriminability: the area under the receiver operating characteristic curve (AUC). Unlike raw differences, SDT metrics such as AUC and d′ can better control the distribution differences by normalizing rating differences using distributional parameters (e.g., the standard deviation in the case of d′). Indeed, under the framework of SDT, discriminability metrics is orthogonal to scaling bias (e.g., the tendency to report higher ratings) (Macmillan & Creelman, 2004). In our study, AUC-based analyses yielded results highly similar to those obtained using our difference-based discriminability metric (Supplementary Figure 4; also see Figure R2 below), suggesting that our findings are robust to potential scaling bias. This point has been made clear in the revised manuscript (lines 743~764).

In addition, we have now explicitly stated in the revised manuscript that our focus is specifically on intensity discriminability, but not discriminability of locations and/or temporal sequences. The title of our manuscript has also been changed to “Neural variability reliably and preferentially encodes *pain intensity discriminability*”. While intensity has long been the center of pain research, some studies have found distinct mechanisms of pain intensity and other aspects of pain such as location (Oshiro et al., 2009). It is thus of interest for future studies to explore whether neural variability also encodes spatial discrimination of pain. These points have been clarified in the revised manuscript (lines 548~552).

**Figure R2. Correlation between neural variability and pain discriminability measured by AUC.**

“2. Clinical Relevance:

The potential clinical relevance of pain discriminability is critical for assessing the overall significance of the findings. The authors cite studies (Ref. 23-26) to support the clinical relevance of their findings, but these studies are somewhat dated and their sample sizes are limited. It would be beneficial for the authors to include more recent studies with larger patient samples. Moreover, it is unclear whether the definitions and operationalizations of pain discriminability in these studies align with those used in the present study. To strengthen the clinical relevance, the authors should ensure that their operationalization of pain discriminability matches the definitions used in previous studies.”

**Reply**: We thank the Reviewer for raising this important concern regarding the clinical relevance of pain discriminability. We acknowledge that some of the earlier studies we cited are relatively dated and based on small samples. This is mainly because there are limited number of studies on pain intensity discrimination. Nonetheless, more recent studies have also found abnormal discriminability in pain patients. For example, Hollins et al. (2012) found that patients with sickle cell disease, which is marked by intense pain, had impaired pain intensity discriminability. Some other studies have found that improving somatosensory discrimination through tactile training could reduce cortical reorganization and pain symptoms in chronic pain patients (Flor et al., 2001; Moseley et al., 2008; Pleger et al., 2005). While not directly related to pain intensity discriminability, these findings suggest a link between discrimination ability and chronic pain. These more recent studies have been cited in support of the clinical relevance of discriminability in the revised manuscript (lines 64~69).

Regarding the alignment of our operationalization of discriminability with previous literature, our primary discriminability measure, rating differences, is conceptually related to SDT-derived metrics of discriminability used in prior studies such as AUC and d′ (Beck et al., 2016; Mancini et al., 2014). In fact, in a control analysis, we have showed that AUC-based analyses yielded results highly similar to those obtained using our difference-based discriminability metric (see Figure R2 above). SDT metrics account for variability in response distributions by normalizing rating differences using a distributional parameter (e.g., the standard deviation in the case of d′). Empirically, we also found that SDT-derived AUC measure strongly correlated with pain rating differences (r = 0.832). Additionally, our previous work demonstrated that the choice of metric (i.e., rating differences, d′, or AUC) did not affect the relationship between pain discriminability and EEG responses (Zhang et al., 2022). Thus, our operationalization of pain discriminability aligns with that used in previous studies, and this point has been clarified in the revised manuscript (lines 749~764).

“3. Independence of Variability and Amplitudes:

A central claim of the study is that neural variability and amplitudes differently encode pain discriminability. This claim is supported by control analyses. However, the significance of these control analyses is not fully explained. Specifically, does subtracting or controlling for differences in mean amplitudes between intensities completely remove amplitude effects, or could it instead reflect differences in signal-to-noise ratio between intensities? To address this, it is essential to demonstrate that the control analyses effectively remove amplitude effects, perhaps through simulations. Additionally, this aspect should be reviewed by an expert in EEG methods to ensure its validity.”

**Reply**: We thank the Reviewer for highlighting this important issue regarding the potential confounding influence of amplitude on our variability-based findings. Actually, our claim that neural variability and amplitude independently encode pain discriminability is supported by four control analyses. First, we used partial correlations to control for ERP amplitude, yet still observed significant correlations between neural variability and pain discriminability. Second, we used permutation entropy as a variability measure, which is less sensitive to ERP amplitude, yielding results consistent with SD-based results. Third, we removed mean ERP waveforms from single-trial EEG data before computing neural variability. Since the mean amplitude of subtracted signals has no variance (i.e., having a constant value of 0), any remaining correlation between EEG variability and discriminability cannot be attributed to amplitude differences. Fourth, frequency band analyses showed that variability-discriminability correlations were preserved in beta, alpha, delta, and theta frequency bands, while the correlation between amplitude and discriminability was only obtained in the delta and theta frequency bands. This divergence indicates that variability-discriminability correlations in higher frequency bands (alpha, beta) could not be attributed to EEG amplitude.

While these findings support the independence of amplitude and variability effects, we acknowledge that amplitude-related confounds, particularly those related to signal-to-noise ratio (SNR), may not be completely eliminated. As suggested, we have conducted simulation analyses to further examine the impact of SNR differences between intensity conditions on our results. Specifically, we sampled noise from baseline (-500ms ~ 0 ms) in single trial EEG data in the low or high intensity conditions. This approach ensures that the noise structure in real EEG signals is respected and that residual noise after preprocessing such as muscle artifacts, baseline drift, and amplifier noise can be captured. The sampled noise was then scaled by a factor (referred to as “noise scale”) and added to post-stimulus EEG signals. Eleven noise scales were chosen: 0~2 in steps of 0.2. Introducing additional noise to the low or high intensity conditions dramatically altered neural variability as measured by SD (Supplementary Figure 6 A&C; see also Figure R3 A&C). Nonetheless, noise seemed to have no substantial effects on our correlation results. Partial correlations between pain intensity discriminability and neural variability while controlling for EEG amplitude differences persisted across various noise levels (Supplementary Figure 6 B&D; see also Figure R3 B&D). Only when an excessive amount of noise (noise scale = 2) was added to the high intensity condition did we obtain no support for the partial correlation. It is noteworthy that this level of noise results in implausible alterations to EEG signal. Furthermore, adding noise to the high and low intensity conditions simultaneously also yielded similar results (Supplementary Figure 6 E&F; see also Figure R3 E&F). Altogether, these simulation analyses suggest that our findings are not artifacts caused by noise differences between intensity conditions.

We have incorporated the noise simulation analyses in the revised manuscript (lines 186~201 and lines 873~884).

**Figure R3. Effects of adding different levels of noise to EEG data on correlations between pain intensity discriminability and neural variability in Dataset 1.**

**(A)** ∆SD time series when different levels of noise were added to the low-intensity condition. As the added noise level increased, SD in the high-intensity condition became smaller than that in the low-intensity condition. Noise scale represents the level of noise added. 11 noise scales (0~2 in steps of 0.2) were tested. A noise scale of *s* means *s* times the baseline noise in single trial EEG signals was added. (**B**) Bayes factor (BF) for partial correlations between pain intensity discriminability and neural variability when noise was added to the low-intensity condition while controlling for ERP amplitude differences. Enclosed areas represent correlations with log_10_(BF) ≥ 0.5, namely, BF ≥ 3.3. Adding noise to the low-intensity condition had no substantial effect on the correlation between pain intensity discriminability and neural variability. (**C**) ∆SD time series when different levels of noise were added to the high-intensity condition. (**D**) BF for partial correlations between pain intensity discriminability and neural variability when different levels of noise were added to the high-intensity condition while controlling for ERP amplitude differences. Adding noise to the high-intensity condition also had no substantial effect on the correlation between pain intensity discriminability and neural variability. (**E**) ∆SD time series when different levels of noise were added to the high- and low-intensity conditions simultaneously. (**F**) BF for partial correlations between pain intensity discriminability and neural variability when different levels of noise were added to the high- and low-intensity conditions while controlling for ERP amplitude differences. Adding noise also had no substantial effect on the correlation between pain intensity discriminability and neural variability.

“4. Comparisons Across Modalities:

One key finding of the study is that neural variability selectively encodes pain discriminability. This conclusion is based on weaker or fewer correlations between neural variability and discriminability in other sensory modalities. However, the difference between modalities has not been statistically tested directly. A direct statistical comparison of the variability-discriminability relationships between modalities is necessary to strengthen this finding. Moreover, the authors should consider whether the slower conduction velocities of nociceptive fibers, compared to other sensory modalities, may lead to higher dispersion and variability in brain responses. This could contribute to differences in signal-to-noise ratio, which might ultimately explain the modality-specific findings.”

**Reply**: We thank the Reviewer for this insightful comment. In response, we have directly compared the correlation coefficients between modalities to statistically assess whether neural variability preferentially encodes pain intensity discriminability. In Dataset 1, direct comparisons between pain and non-pain modalities showed significantly stronger correlations for pain: (1) pain vs. touch: z = 2.95, p = 0.003; (2) pain vs. audition: z = 2.91, p = 0.004; (3) pain vs. vision: z = 3.68, p = 0.0002. In Datasets 2&3, direct comparisons between pain and non-pain modalities also indicated preferential encoding of pain intensity discriminability: (1) pain vs. touch: z = 4.12, p = 0.00004; (2) pain vs. audition: z = 3.96, p = 0.00007; (3) pain vs. vision: z = 3.86, p = 0.0001. After matching subjective rating across modalities, our conclusion also largely held when directly comparing pain and non-pain modalities. In Dataset 1: (1) pain vs. touch: z = 2.09, p = 0.037; (2) pain vs. audition: z = 0.96, p = 0.34; (3) pain vs. vision: z = 2.93, p = 0.003. In Datasets 2&3: (1) pain vs. touch: z = 3.18, p = 0.001; (2) pain vs. audition: z = 2.16, p = 0.031; (3) pain vs. vision: z = 4.01, p = 0.00006. These additional analyses have been incorporated into the revised manuscript (lines 287~298, lines 317~330).

To further reinforce our findings, as suggested by Reviewer 3, we have also computed Bayes factors (BF). The BF-based results align well with our original findings and support the conclusion that neural variability more robustly encodes pain discriminability (Figures 2&5; also see Figures R4&R5 below).

Regarding the concern about conduction velocity differences, we have conducted simulation analyses to assess whether differences in signal-to-noise ratio (SNR), potentially arising from slower nociceptive conduction velocities of nociceptive fibers, could account for our findings. The results showed that the correlations between neural variability and pain discriminability remained robust across a wide range of added noise levels (see Figure R3 above). Only when excessive noise was added did the correlation diminish, suggesting that such an effect would require implausibly high noise levels. Notably, one reason why slower conduction velocities may influence SNR of averaged ERPs is latency jitter of single trial ERPs (Luck, 2014). However, this mechanism mainly affects averaged ERP responses, not in single-trial measures like temporal SD. Importantly, our temporal SD metric was computed from windowed (100 ms), single-trial EEG responses rather than averaged ERPs. Therefore, the modality-specific findings in the present study seem unlikely to be influenced by the reduced SNR due to slower conduction velocities of nociceptive fibers relative to non-pain modalities.

**Figure R4. Neural variability reliably correlated with pain discriminability as assessed by Bayes factor.** The gray-shaded bars represent Pearson’s r values at time points where significant correlations were observed after FDR correction. The BF color bars underneath display the corresponding Bayes Factor values for the correlations.

**Figure R5. Neural variability of EEG responses evoked by tactile, auditory, and visual stimuli did not reflect their respective sensory intensity discriminability.**

**(A−C)** Partial correlations between neural variability and sensory intensity discriminability of tactile, auditory, and visual stimuli in Dataset 1. See point-to-point partial correlations in Supplementary Figure 9. No significance was observed for all non-pain modalities in partial correlations while controlling for mean amplitude differences of ERPs. **(D−F)** Partial correlations between neural variability and sensory intensity discriminability in Datasets 2&3. No significant partial correlations while controlling for mean amplitude differences were observed for any of these sensory modalities. The *r* shown in panels represent r values of partial correlation. BF is short for Bayes Factor.

“5. Comparisons Across Groups:

Another key finding is that the relationship between neural variability and pain discriminability was partly disrupted in patients with chronic pain. This conclusion is based on weaker correlations in patients compared to healthy controls. However, the sample size of the patient group is relatively small (N=27), and the difference between groups has not been directly tested. A statistical comparison of the variability-discriminability relationships between the two groups is necessary to substantiate this claim.”

**Reply**: We thank the Reviewer for this valuable comment. While we observed differences in the strength of the neural variability–pain discriminability relationship between healthy controls and patients with chronic pain, our conclusion was drawn from within-subject comparisons between affected and unaffected sides in patients with chronic pain. We believe direct comparisons between patients and healthy controls are not appropriate due to substantial differences in datasets and demographics. We have clarified this rationale in the revised manuscript (lines 344~347).

Additionally, given the relatively small sample size in the patient group (N = 27) and insufficient statistical power, we refrained from statistically comparing the variability-discriminability relationships between affected and unaffected sides. Testing the difference between two correlation coefficients generally requires larger sample size to achieve adequate statistical power. In small samples, such comparisons are particularly vulnerable to Type II errors and limited generalizability (Button et al., 2013). Nevertheless, to strengthen confidence in our findings, we complemented our frequentist analyses with Bayesian statistics. Our original results from frequentist statistics were largely confirmed by Bayesian statistics, especially for the comparisons between the 4.25J vs 3.75J and 4.25 J vs 4.00J (Figure 8; also Figure R6 below). These converging results add weight to our conclusion that the relationship between neural variability and pain discriminability may be altered in chronic pain.

**Figure R6. Neural variability as an indicator of pain intensity discriminability in Dataset 6 (chronic pain patients) with multiple levels of stimulus intensities.**

**(A)** Pain intensity ratings (left panel), temporal SD in the unaffected and affected sides (middle two panels), and P values of paired-sample t-tests for temporal SD between pairs of stimulus intensities (right panel) in Dataset 6 (PHN patients, N = 27). Paired-sample t-tests revealed significant differences for most stimulus pairs with a difference exceeding 0.5 J on both sides. Significance was highlighted in green for the unaffected side and red for the affected side (left panel). Temporal SDs showed significant differences in all pairs of stimulus intensities for both sides (right panel). **(B-C)** Correlations between ∆SD and pain intensity discriminability were assessed for both unaffected side (B) and affected side (C). Significant correlations were observed in four pairs of intensities on the unaffected side and none pair of intensities on the affected side, indicating a disruption of the correlations in the affected side. BF is short for Bayes Factor. Error bars in A and C are standard errors of means, and error bars in B and D represent 95% confidence intervals.

“6. FDR Corrections:

The methods section should provide more clarity on how false discovery rate (FDR) corrections were performed. Specifically, were the corrections applied across time for each electrode, or were they applied across both time and space (i.e., across electrodes)?”

**Reply**: We applied FDR corrections across time for electrode Cz only, as this was our primary electrode of interest. Topographical maps are presented for illustrative purposes only. No statistical inferences were made across electrodes, and our conclusions do not depend on spatial distributions. This point has been clarified in the revised manuscript (lines 781~784). Please note that we have also computed BF to reinforce our original conclusions based on frequentist statistics. The BF-based results aligned well with our original findings, supporting the reliability of our results.

**Reviewer #3:**

This manuscript presents an extensive analysis of EEG data to investigate how neural variability relates to "pain discriminability." The study spans multiple large datasets and provides a compelling case for considering neural variability—measured as temporal standard deviation (SD)—as an independent contributor to how pain is processed in the brain. While the findings are robust and the dataset impressive, several conceptual and methodological concerns should be addressed to strengthen the claims.

**Reply**: We thank the reviewer for their positive feedback and valuable suggestions. We provide a point-to-point reply to the comments in the following.

“METHODOLOGY

1. Choice of Variability Metric

The use of temporal standard deviation (SD) as the primary measure of neural variability raises important concerns. SD is an absolute measure and is inherently tied to the scale of the signal. This makes it problematic for comparisons across participants, conditions, modalities, and datasets, particularly when the mean EEG amplitude varies. A more appropriate and statistically rigorous approach to consider would be to use the coefficient of variation (CV)—the ratio of the standard deviation to the mean—which is a normalized measure and allows for comparisons that account for differences in signal magnitude. This would help to disentangle variability due to inherent neural dynamics from those merely reflecting changes in mean amplitude.”

**Reply**: We appreciate the Reviewer’s thoughtful comment regarding the scale-dependency of SD as a measure of neural variability. While we acknowledge that SD is an absolute metric and thus sensitive to differences in signal magnitude, we note that the coefficient of variation (CV), obtained by normalizing for mean signal magnitude, becomes unstable when the mean approaches zero. We illustrate this instability in Supplementary Figure 10 (also see Figure R7 below). Given the volatile behavior of CV, we chose to use SD and permutation entropy (PE), both of which are commonly accepted in the field (Waschke et al., 2021). This rationale has been explained in the revised manuscript (lines 712~716).

To mitigate the potential confounding influence of amplitude, we conducted several control analyses. First, we used partial correlations to control for ERP amplitude, yet still observed significant correlations between neural variability and pain discriminability. Second, we used permutation entropy as a variability measure, which only retains the order relations between values in the time series rather than their absolute numerical values (Bandt & Pompe, 2002), thus less affected by signal scaling and yielded results consistent with SD-based results. Third, we removed mean ERP waveforms from single-trial EEG data before computing neural variability. Since the mean amplitude of subtracted signals has no variance (i.e., having a constant value of 0), any remaining correlation between EEG variability and discriminability cannot be attributed to amplitude differences. Fourth, frequency band analyses showed that variability-discriminability correlations were preserved in beta, alpha, delta, and theta frequency bands, while the correlation between amplitude and discriminability was only obtained in the delta and theta frequency bands. This divergence indicates that variability-discriminability correlations in higher frequency bands (alpha, beta) could not be attributed to EEG amplitude. Results from these analyses consistently support the independence of variability from amplitude, and we have expanded our discussion of these control analyses in the revised manuscript (lines 437~448, and lines 459~465).

**Figure R7. Instability of CV as a measure of neural variability of pain in Dataset 1.** Note that the y axis is capped at ±150 for better visualization. Actually, CV values ranged from a maximum of 2442 to a minimum of -5017, illustrating its instability in conditions with low mean signal amplitudes.

“2. Single-Trial Measures - Practical Limitations and Interpretability

The authors highlight that one key advantage of neural variability (e.g., temporal SD) is its ability to be computed at the single-trial level, unlike ERP amplitude, which generally requires trial averaging to achieve a usable signal-to-noise ratio. While this is technically true, the practical utility and interpretability of such single-trial measures deserve more scrutiny.

For example, let's say a 100-ms sliding window is used to calculate SD. At a sampling rate of 1,000 Hz (as in the study), this gives only 100 data points per window. If there's residual noise (e.g., muscle artifacts, baseline drift, or amplifier noise), that noise directly inflates the SD measure at the trial level. This is particularly problematic in EEG, where the variance of the noise can easily approach or exceed the variance of the signal, especially in higher frequencies. Moreover, SD is inherently sensitive to nonstationarity. For example, a transient high-amplitude burst (e.g., from a blink or movement) may drastically inflate SD in one window but not others—even if such an artifact does not reflect neural variability per se.

The authors could provide an estimate of the signal-to-noise ratio of their SD or other variability measurements at the single-trial level and demonstrate robustness (e.g., test-retest reliability or within-subject stability across blocks).”

**Reply**: We appreciate the reviewer’s insightful comments, and we acknowledge that single-trial SD is susceptible to residual noise even after the low-pass filtering (<30 Hz). We note, however, it is possible to compute SD from the trial-averaged EEG data, which are inherently less noisy than single trial data since residual noise would be averaged out. We computed SD from trial-averaged data and observed comparable results to those obtained from single-trial data (Supplemental Figure 6; also see Figure R8 below). Comparisons between single trial- and average trial-SD have been added in the revised manuscript (lines 182~186).

Moreover, as suggested by the Reviewer and also Reviewer 2, we have performed a simulation analysis to quantify the influence of the signal-to-noise ratio of EEG responses on our variability metrics. Specifically, we sampled noise from baseline (-500ms ~ 0 ms) in single trial EEG data in the low or high intensity conditions. This approach ensures that the noise structure in real single trial EEG data is respected and that residual noise after preprocessing such as muscle artifacts, baseline drift, and amplifier noise can be captured. The sampled noise was then scaled by a factor (referred to as “noise scale”) and added to post-stimulus EEG signals. Eleven noise scales were chosen: 0~2 in steps of 0.2. Introducing additional noise to the low or high intensity conditions dramatically altered neural variability as measured by SD (Supplementary Figure 6 A&C; see also Figure R9 A&C). Nonetheless, noise seemed to have no substantial effects on our correlation results. Partial correlations between pain intensity discriminability and neural variability while controlling for EEG amplitude differences persisted across various noise levels (Supplementary Figure 6 B&D; see also Figure R9 B&D). Only when an excessive amount of noise (noise scale = 2) was added to the high intensity condition did we obtain no support for the partial correlation. It is noteworthy that this level of noise results in implausible alterations to real EEG signal. Furthermore, adding noise to the high- and low-intensity conditions simultaneously also yielded similar results (Supplementary Figure 6 E&F; see also Figure R9 E&F). Altogether, these simulation analyses suggest that our findings are not artifacts caused by noise differences between intensity conditions. Noise simulation analyses have been added in the revised manuscript (lines 186~201 and lines 873~884).

The sensitivity of SD to nonstationarity was also partly addressed in our control analysis by including permutation entropy as a complementary neural variability metric (Supplementary Figure 6; also see Figure R10 below). Permutation entropy depends on only the ordinal relations between values in the time series rather than their absolute numerical values, and is thus less affected by transient high-amplitude burst (Bandt & Pompe, 2002).

Together, these additional control and simulation analyses support the validity and reliability of our main conclusions.

**Figure R8. Comparable results from single trial-based and average trial-based analyses for pain in Dataset 1.**

**Figure R9. Effects of adding different levels of noise to EEG data on correlations between pain intensity discriminability and neural variability in Dataset 1.**

**(A)** ∆SD time series when different levels of noise were added to the low-intensity condition. As the added noise level increased, SD in the high-intensity condition became smaller than that in the low-intensity condition. Noise scale represents the level of noise added. 11 noise scales (0~2 in steps of 0.2) were tested. A noise scale of *s* means *s* times the baseline noise in single trial EEG signals was added. (**B**) Bayes Factor (BF) for partial correlations between pain intensity discriminability and neural variability when noise was added to the low-intensity condition while controlling for ERP amplitude differences. Enclosed areas represent correlations with log_10_(BF) ≥ 0.5, namely, BF ≥ 3.3. Adding noise to the low-intensity condition had no substantial effect on the correlation between pain intensity discriminability and neural variability. (**C**) ∆SD time series when different levels of noise were added to the high-intensity condition. (**D**) BF for partial correlations between pain intensity discriminability and neural variability when different levels of noise were added to the high-intensity condition while controlling for ERP amplitude differences. Adding noise to the high-intensity condition also had no substantial effect on the correlation between pain intensity discriminability and neural variability. (**E**) ∆SD time series when different levels of noise were added to the high- and low-intensity conditions simultaneously. (**F**) BF for partial correlations between pain intensity discriminability and neural variability when different levels of noise were added to the high- and low-intensity conditions while controlling for ERP amplitude differences. Adding noise also had no substantial effect on the correlation between pain intensity discriminability and neural variability.

**Figure R10. Information theory-based neural variability encodes pain intensity discriminability in Dataset 1.**

**(A)** Neural variability quantified by an information theory-based measure, temporal PE of EEG responses evoked by high- (violet) and low-intensity (gold) stimuli in Dataset 1. **(B)** Point-by-point correlations between neural variability differences (high – low, ∆PE) and rating differences (high – low, ∆Rating). Note that the gray bars represent Pearson’s r values at time points where significant correlations were observed after FDR correction. The color bars underneath display the corresponding Bayes Factor values. **(C)** Correlations between ∆Rating and PE values averaged around the trough of temporal PE. Error bars are 95% confidence intervals. BF is short for Bayes Factor.

“3. Multiplicity and Overfitting

The manuscript involves a massive number of statistical comparisons, including:

* Point-by-point correlations across time (on the order of hundreds per electrode).

* Multiple datasets.

* Multiple metrics of variability (SD, permutation entropy).

* Multiple time windows (50 ms, 100 ms, 200 ms).

* Multiple outcome variables (pain discriminability, intraindividual perception, sensitivity).

* Different filtering bands (delta, theta, alpha, beta).

While some corrections (e.g., FDR) are applied, the overall analytical flexibility introduces serious risks of overfitting and false positives.

The authors could consider a cross-validation procedure (e.g., split-half analysis or leave-one-subject-out) to validate the generalizability of the correlations. Alternatively, they could report Bayes factors, which can help convey robustness beyond binary significance testing.”

**Reply**: We appreciate the reviewer’s concern regarding analytical flexibility and the associated risks of overfitting and false positives. The cross-validation approach is a powerful way to test the generalizability of our findings. Nevertheless, the lack of a formal predictive model in our study makes it less appropriate to do cross-validation on our data. Instead, we consider the replication of results across independent datasets as an informal but meaningful validation, since different datasets were analyzed separately. To further confirm our findings, we computed Bayes factors for all analyses, as recommended. BF-based results complement the frequentist statistics and help quantify the strength of evidence beyond binary significance thresholds. Importantly, the BF-based results confirmed our conclusions drawn from the original analyses (see Figures R11&R12 below for examples).

**Figure R11. Neural variability reliably correlated with pain discriminability as assessed by Bayes factor.** The gray-shaded bars represent Pearson’s r values at time points where significant correlations were observed after FDR correction. The BF color bars underneath display the corresponding Bayes Factor values for the correlations.

**Figure R12. Neural variability of EEG responses evoked by tactile, auditory, and visual stimuli did not reflect their respective sensory intensity discriminability.**

**(A−C)** Partial correlations between neural variability and sensory intensity discriminability of tactile, auditory, and visual stimuli in Dataset 1. See point-to-point partial correlations in Supplementary Figure 9. No significance was observed for all non-pain modalities in partial correlations while controlling for mean amplitude differences of ERPs. **(D−F)** Partial correlations between neural variability and sensory intensity discriminability in Datasets 2&3. No significant partial correlations while controlling for mean amplitude differences were observed for any of these sensory modalities. The *r* shown in panels represent r values of partial correlation. BF is short for Bayes Factor.

“4. Clarity in Data Presentation

It is not clearly stated in the main text or figure captions whether the point-by-point correlations shown in figures (e.g., between neural variability and pain discriminability) are computed at a specific EEG electrode or represent averages across a region or electrode cluster. If a specific electrode was used—such as Cz or Pz—this needs to be stated explicitly. If averages were taken over a region (e.g., central electrodes), that too should be clarified, including which electrodes were included. This information is crucial for interpreting the spatial specificity of the reported effects.”

**Reply**: We confirm that point-by-point correlations were computed at electrode Cz only, as this was our primary electrode of interest. This information has been clearly stated in the revised manuscript (lines 782~784).

“CONCEPT & INTERPRETATION

5. Definition of "Sensory Discriminability"

The term "sensory discriminability" is somewhat misleading in this context. Traditionally, this term implies a measure of perceptual discrimination ability—such as the just-noticeable difference or psychophysical thresholds. However, in this study, "discriminability" is operationalized as the difference in mean subjective ratings between low- and high-intensity stimuli. This metric is highly sensitive to factors such as arousal, alertness, attentional engagement, and stimulus surprise—not just sensory processing per se. It is essential that this distinction be made explicit early in the manuscript to avoid confusion and to properly frame the interpretation of the results.

**Reply**: Just-notifiable difference (JND) is indeed the traditional metric for quantifying discriminability. However, we reason that JND and rating differences can be conceptualized as two distinct operationalizations of discriminability. Conceptually, JND holds constant pain sensation (i.e., just noticeable difference in perception) and uses the difference of physical intensities between two stimuli to quantify the ability of individuals to discriminate between them. On the other hand, rating difference fixes the physical stimulus intensities and quantifies pain discriminability as the difference in subjective pain ratings between the two stimuli. The fix-intensity approach has two practical advantages: (1) it is practically easier to fix stimulus intensity than to control subjective perception, and (2) the limited precision of laser intensity adjustment (e.g., 0.25J step size in the laser stimulator we used) can make JND measurements less reliable. With these considerations in mind, we believe it’s reasonable to quantify discriminability using rating differences in our context. As suggested by the Reviewer, we have made explicit the distinction between JND and rating differences in the revised manuscript to avoid confusion (lines 109~121).

Raw rating differences, however, do not fully account for internal scaling of pain intensity and/or individual variations in using rating scales. To address this concern, we conducted control analyses using a signal detection theory (SDT)-based, non-parametric measure of discriminability: the area under the receiver operating characteristic curve (AUC). Unlike raw rating differences, SDT metrics such as AUC and d′ can better control distribution differences in ratings by normalizing rating differences using distributional parameters (e.g., standard deviation in the case of d′). Indeed, under the framework of SDT, discriminability metrics is orthogonal to scaling bias (e.g., the tendency to report higher ratings) (Macmillan & Creelman, 2004). In our study, AUC-based analyses yielded results highly similar to those obtained using our difference-based discriminability metric (Supplementary Figure 4; also see Figure R13 below), suggesting that our findings are robust to potential scaling bias. This point has been made clear in the revised manuscript (lines 743~764).

**Figure R13. Correlation between neural variability and pain discriminability measured by AUC.**

6. Interpretation: Attention vs. Sensory Specificity

Given that the discriminability metric used likely reflects attentional modulation more than pure sensory encoding, it is perhaps not surprising that the effects are most prominent in the nociceptive modality. Painful stimuli are inherently salient and demand more attentional resources than nonpainful stimuli. Thus, the observed selectivity for pain may reflect the greater attentional capture of nociceptive input rather than a specific pain-related neural mechanism. This should be more clearly discussed in the manuscript, possibly reframing "selectivity for pain" as "selectivity for salient or attention-grabbing stimuli" (or something along these lines)

**Reply**: We agree that attentional modulation is an important alternative interpretation of our findings. However, our comparisons between pain and non-pain modalities may provide some indirect evidence against a purely attentional account, since all transient sensory stimuli are inherently salient and attention-grabbing (Legrain et al., 2011). Indeed, similar neural responses across pain and non-pain sensory modalities are one of the main arguments for the salience-focused interpretation (Mouraux et al., 2011; Mouraux & Iannetti, 2009, 2018). Notably, intensity ratings have also been shown to highly correlate with stimulus salience (e.g., r = 0.84 in Liang et al., 2019 and r = 0.93 in Hadjis et al., 2025). Our result that neural variability correlated with pain intensity discriminability after matching intensity ratings thus indirectly suggests that salience may not have substantially affected our findings. However, it remains possible that painful stimuli may command greater attention than equally intense nonpainful stimuli. Since no salience ratings or physiological signals of arousal like skin conductance responses were collected (Hadjis et al., 2025), it is impossible to directly test the influence of salience on our findings and rule out this alternative interpretation. Further research needs to directly examine the role of attention in our findings. We have discussed these points in the revised manuscript (lines 509~525).

To avoid confusion and overinterpretation of our findings, we have replaced the term “pain selectivity” with the more neutral “pain preferential” throughout the manuscript. Additionally, we have rephrased the third key question our study attempted to answer as whether neural variability reflects *stimulus-general* factors without specifying salience or modality selectivity. Given the different findings in pain and non-pain modalities, we believe it is more justifiable to describe the association between neural variability and pain intensity discriminability as not reflecting a stimulus-general mechanism.

7. Terminology: "Pain Selectivity"

The term "pain selectivity" is used frequently but may overstate the findings. Since nociceptive stimuli are inherently more salient and attentionally engaging, as discussed, the observed selectivity could reflect differences in attentional salience rather than sensory modality specificity. The authors might consider using a more neutral term unless specificity can be more rigorously demonstrated.”

**Reply**: We appreciate the reviewer’s thoughtful comment. In response, and as noted in our reply to the previous comment, we have replaced the term **"pain selectivity"** with the more neutral term **"pain preferential"** throughout the manuscript. While less conventional, this terminology better reflects the observed effects without implying strict sensory specificity. Additionally, we have rephrased the third key question our study attempted to answer as whether neural variability reflects *stimulus-general* factors, without specifying salience.

“8. Theoretical Framing: Bayesian Inference and Predictive Coding

The findings align well with theories of Bayesian brain function and predictive coding, where variability in neural responses may reflect uncertainty or the precision of predictions. In these models, increased neural variability can signal greater perceptual uncertainty or shifts in attentional weighting. If they wish, the authors could consider discussing their findings in relation to this broader theoretical framework, which may provide a richer interpretation of their results. Several studies in the predictive coding and Bayesian neuroscience literature could be referenced to strengthen this link.”

**Reply**: We thank the reviewer for this great suggestion. It is true that neural variability reflects the precision of pain predictions. In accord with the Bayesian brain hypothesis, pain perception can be theorized as a Bayesian inference process, which posits that perceived pain is the combination of bottom-up nociceptive inputs and top-down pain predictions weighted by their relative precision (Tabor et al., 2017). This framework has received increasing empirical support across pain and other sensory modalities (Coll et al., 2024; de Lange et al., 2018; Mulders et al., 2023; Onysk et al., 2024). From this perspective, neural variability could indeed reflect perceptual uncertainty or shifts in attentional weighting in the prediction of pain. Consequently, differential neural variability in the high and low intensity conditions may signal varying precision of predictions in different conditions, which then results in change in pain intensity discriminability. This interpretation aligns with previous studies linking neural variability to perceptual uncertainty (Echeveste et al., 2020; Orbán et al., 2016; Skowron et al., 2025) and recent work demonstrating correlations between moment-to-moment variability of BOLD signals and perceptual precision in a learning task (Skowron et al., 2025). Although uncertainty in pain predictions has been related to nociceptive-evoked ERP responses (Mulders et al., 2023), there remains a lack of pain research that directly investigating the relationship between pain prediction precision and neural variability. Future studies are thus in need to directly test the role of pain prediction precision in the association between neural variability and pain intensity discrimination. These points have been discussed in the revised manuscript (lines 491~507).

**References:**

Arazi, A., Censor, N., & Dinstein, I. (2017). Neural Variability Quenching Predicts Individual Perceptual Abilities. *Journal of Neuroscience*, *37*(1), 97–109. https://doi.org/10.1523/JNEUROSCI.1671-16.2016

Bandt, C., & Pompe, B. (2002). Permutation Entropy: A Natural Complexity Measure for Time Series. *Physical Review Letters*, *88*(17), 174102. https://doi.org/10.1103/PhysRevLett.88.174102

Beck, B., Làdavas, E., & Haggard, P. (2016). Viewing the body modulates both pain sensations and pain responses. *Experimental Brain Research*, *234*(7), 1795–1805. https://doi.org/10.1007/s00221-016-4585-9

Bromm, B., & Treede, R.-D. (1987). Human cerebral potentials evoked by CO2 laser stimuli causing pain. *Experimental Brain Research*, *67*(1), 153–162. https://doi.org/10.1007/BF00269463

Button, K. S., Ioannidis, J. P., Mokrysz, C., Nosek, B. A., Flint, J., Robinson, E. S., & Munafò, M. R. (2013). Power failure: Why small sample size undermines the reliability of neuroscience. *Nature Reviews Neuroscience*, *14*(5), 365–376.

Coll, M.-P., Walden, Z., Bourgoin, P.-A., Taylor, V., Rainville, P., Robert, M., Nguyen, D. K., Jolicoeur, P., & Roy, M. (2024). Pain reflects the informational value of nociceptive inputs. *PAIN*, *165*(10), e115. https://doi.org/10.1097/j.pain.0000000000003254

de Lange, F. P., Heilbron, M., & Kok, P. (2018). How do expectations shape perception? *Trends in Cognitive Sciences*. https://doi.org/10.1016/j.tics.2018.06.002

Dinstein, I., Heeger, D. J., & Behrmann, M. (2015). Neural variability: Friend or foe? *Trends in Cognitive Sciences*, *19*(6), 322–328. https://doi.org/10.1016/j.tics.2015.04.005

Echeveste, R., Aitchison, L., Hennequin, G., & Lengyel, M. (2020). Cortical-like dynamics in recurrent circuits optimized for sampling-based probabilistic inference. *Nature Neuroscience*, *23*(9), 1138–1149. https://doi.org/10.1038/s41593-020-0671-1

Flor, H., Denke, C., Schaefer, M., & Grüsser, S. (2001). Effect of sensory discrimination training on cortical reorganisation and phantom limb pain. *The Lancet*, *357*(9270), 1763–1764. https://doi.org/10.1016/S0140-6736(00)04890-X

Garrett, D. D., Samanez-Larkin, G. R., MacDonald, S. W. S., Lindenberger, U., McIntosh, A. R., & Grady, C. L. (2013). Moment-to-moment brain signal variability: A next frontier in human brain mapping? *Neuroscience & Biobehavioral Reviews*, *37*(4), 610–624. https://doi.org/10.1016/j.neubiorev.2013.02.015

Gil Ávila, C., Bott, F. S., Tiemann, L., Hohn, V. D., May, E. S., Nickel, M. M., Zebhauser, P. T., Gross, J., & Ploner, M. (2023). DISCOVER-EEG: An open, fully automated EEG pipeline for biomarker discovery in clinical neuroscience. *Scientific Data*, *10*(1), 613. https://doi.org/10.1038/s41597-023-02525-0

Hadjis, G. E., Atlas, L. Y., Mouseli, P., Sexton, C. A., McAndrews, M. P., & Moayedi, M. (2025). Subjective salience ratings are a reliable proxy for physiological measures of arousal. *PAIN*, 10.1097/j.pain.0000000000003699. https://doi.org/10.1097/j.pain.0000000000003699

Hollins, M., Stonerock, G. L., Kisaalita, N. R., Jones, S., Orringer, E., & Gil, K. M. (2012). Detecting the Emergence of Chronic Pain in Sickle Cell Disease. *Journal of Pain and Symptom Management*, *43*(6), 1082–1093. https://doi.org/10.1016/j.jpainsymman.2011.06.020

Kloosterman, N. A., Kosciessa, J. Q., Lindenberger, U., Fahrenfort, J. J., & Garrett, D. D. (2020). Boosts in brain signal variability track liberal shifts in decision bias. *eLife*, *9*, e54201. https://doi.org/10.7554/eLife.54201

Legrain, V., Iannetti, G. D., Plaghki, L., & Mouraux, A. (2011). The pain matrix reloaded: A salience detection system for the body. *Progress in Neurobiology*, *93*(1), 111–124.

Li, Z., Zhang, L., Zeng, Y., Zhao, Q., & Hu, L. (2023). Gamma-band oscillations of pain and nociception: A systematic review and meta-analysis of human and rodent studies. *Neuroscience & Biobehavioral Reviews*, *146*, 105062. https://doi.org/10.1016/j.neubiorev.2023.105062

Liang, M., Su, Q., Mouraux, A., & Iannetti, G. D. (2019). Spatial patterns of brain activity preferentially reflecting transient pain and stimulus intensity. *Cerebral Cortex*, *29*(5), 2211–2227. https://doi.org/10.1093/cercor/bhz026

Luck, S. J. (2014). *An Introduction to the Event-Related Potential Technique* (2nd ed.). MIT Press.

Macmillan, N. A., & Creelman, C. D. (2004). *Detection Theory: A User’s Guide*. Psychology Press.

Mancini, F., Nash, T., Iannetti, G. D., & Haggard, P. (2014). Pain relief by touch: A quantitative approach. *Pain*, *155*(3), 635–642. https://doi.org/10.1016/j.pain.2013.12.024

May, E. S., Tiemann, L., Gil Ávila, C., Bott, F. S., Hohn, V. D., Gross, J., & Ploner, M. (2025). Assessing the predictive value of peak alpha frequency for the sensitivity to pain. *Pain*. https://doi.org/10.1097/j.pain.0000000000003571

McIntosh, A. R., Kovacevic, N., & Itier, R. J. (2008). Increased Brain Signal Variability Accompanies Lower Behavioral Variability in Development. *PLOS Computational Biology*, *4*(7), e1000106. https://doi.org/10.1371/journal.pcbi.1000106

Moseley, G. L., Zalucki, N. M., & Wiech, K. (2008). Tactile discrimination, but not tactile stimulation alone, reduces chronic limb pain. *Pain*, *137*(3), 600–608. https://doi.org/10.1016/j.pain.2007.10.021

Mouraux, A., Diukova, A., Lee, M. C., Wise, R. G., & Iannetti, G. D. (2011). A multisensory investigation of the functional significance of the “pain matrix.” *NeuroImage*, *54*(3), 2237–2249.

Mouraux, A., & Iannetti, G. D. (2009). Nociceptive laser-evoked brain potentials do not reflect nociceptive-specific neural activity. *Journal of Neurophysiology*, *101*(6), 3258–3269.

Mouraux, A., & Iannetti, G. D. (2018). The search for pain biomarkers in the human brain. *Brain*, *141*(12), 3290–3307. https://doi.org/10.1093/brain/awy281

Mulders, D., Seymour, B., Mouraux, A., & Mancini, F. (2023). Confidence of probabilistic predictions modulates the cortical response to pain. *Proceedings of the National Academy of Sciences*, *120*(4), e2212252120. https://doi.org/10.1073/pnas.2212252120

Mullen, T. R., Kothe, C. A. E., Chi, Y. M., Ojeda, A., Kerth, T., Makeig, S., Jung, T.-P., & Cauwenberghs, G. (2015). Real-time neuroimaging and cognitive monitoring using wearable dry EEG. *IEEE Transactions on Biomedical Engineering*, *62*(11), 2553–2567. https://doi.org/10.1109/tbme.2015.2481482

Nickel, M. M., Tiemann, L., Hohn, V. D., May, E. S., Ávila, C. G., Eippert, F., & Ploner, M. (2022). Temporal–spectral signaling of sensory information and expectations in the cerebral processing of pain. *Proceedings of the National Academy of Sciences*, *119*(1). https://doi.org/10.1073/pnas.2116616119

Onysk, J., Gregory, N., Whitefield, M., Jain, M., Turner, G., Seymour, B., & Mancini, F. (2024). Statistical learning shapes pain perception and prediction independently of external cues. *eLife*, *12*. https://doi.org/10.7554/eLife.90634.2

Orbán, G., Berkes, P., Fiser, J., & Lengyel, M. (2016). Neural Variability and Sampling-Based Probabilistic Representations in the Visual Cortex. *Neuron*, *92*(2), 530–543. https://doi.org/10.1016/j.neuron.2016.09.038

Oshiro, Y., Quevedo, A. S., McHaffie, J. G., Kraft, R. A., & Coghill, R. C. (2009). Brain mechanisms supporting discrimination of sensory features of pain: A new model. *Journal of Neuroscience*, *29*(47), 14924–14931. https://doi.org/10.1523/JNEUROSCI.5538-08.2009

Pion-Tonachini, L., Kreutz-Delgado, K., & Makeig, S. (2019). ICLabel: An automated electroencephalographic independent component classifier, dataset, and website. *NeuroImage*, *198*, 181–197. https://doi.org/10.1016/j.neuroimage.2019.05.026

Pleger, B., Tegenthoff, M., Ragert, P., Förster, A.-F., Dinse, H. R., Schwenkreis, P., Nicolas, V., & Maier, C. (2005). Sensorimotor returning in complex regional pain syndrome parallels pain reduction. *Annals of Neurology*, *57*(3), 425–429. https://doi.org/10.1002/ana.20394

Schurger, A., Sarigiannidis, I., Naccache, L., Sitt, J. D., & Dehaene, S. (2015). Cortical activity is more stable when sensory stimuli are consciously perceived. *Proceedings of the National Academy of Sciences*, *112*(16), E2083–E2092. https://doi.org/10.1073/pnas.1418730112

Skowron, A., Kosciessa, J. Q., Lorenz, R., Hertwig, R., Bos, W. van den, & Garrett, D. D. (2025). *Neural variability compresses with increasing belief precision during Bayesian inference* (p. 2024.01.11.575180). bioRxiv. https://doi.org/10.1101/2024.01.11.575180

Tabor, A., Thacker, M. A., Moseley, G. L., & Körding, K. P. (2017). Pain: A statistical account. *PLOS Computational Biology*, *13*(1), e1005142. https://doi.org/10.1371/journal.pcbi.1005142

Valentini, E., Shindy, A., Witkovsky, V., Stankewitz, A., & Schulz, E. (2023). Interindividual Variability and Individual Stability of Pain- and Touch-Related Neuronal Gamma Oscillations. *Journal of Neurophysiology*. https://doi.org/10.1152/jn.00530.2021

Waschke, L., Kloosterman, N. A., Obleser, J., & Garrett, D. D. (2021). Behavior needs neural variability. *Neuron*, *109*(5), 751–766. https://doi.org/10.1016/j.neuron.2021.01.023

Zhang, L.-B., Lu, X.-J., Huang, G., Zhang, H.-J., Tu, Y.-H., Kong, Y.-Z., & Hu, L. (2022). Selective and replicable neuroimaging-based indicators of pain discriminability. *Cell Reports Medicine*, *3*(12), 100846. https://doi.org/10.1016/j.xcrm.2022.100846
