# Supplementary material for: Neural variability reliably encodes interindividual differences in the perception of pain intensity
Source: PLoS Biol. 2025 Oct 27;23(10):e3003470. doi: 10.1371/journal.pbio.3003470 (PMC12574952; doi:10.1371/journal.pbio.3003470)
Supplement: Attachment — Submitted filename: Response to Reviewers-rev2-F.docx [file pbio.3003470.s014.docx]

**Reviewer #1:**

General Comments:
The authors have made commendable efforts to address the concerns raised in the initial review. They have performed numerous additional control analyses, which have considerably strengthened the manuscript. The replies to my previous comments are thoughtful and mostly convincing, and overall, the manuscript is now much improved in clarity and rigor.

A few issues remain where I believe further clarification or moderation would improve the paper. These relate to the interpretation of modality specificity, the strength and clinical relevance of the patient findings, the definition and interpretation of discriminability, and the scope of what the results mean.

**Reply**: We appreciate the reviewer’s thoughtful and constructive comments that improved our manuscript. In response to the reviewer’s remaining concerns, we have revised our manuscript accordingly and provided detailed point-by-point replies below.

Specific Comments:
1. Specificity of pain-related discriminability
The authors argue for a modality-specific role of neural variability in pain discriminability. While the new analyses are convincing, I still find it conceptually difficult to believe that fundamentally different mechanisms underlie discriminability for pain compared to other sensory modalities. I do not see a straightforward way to further test this empirically with the present dataset, but it may help if the discussion more fully considers this phenomenon, including alternative interpretations.

**Reply**: We agree that there is not clear reason why pain intensity discriminability, but not non-pain intensity discriminability, seems to be encoded by neural variability. We discussed the potential influence of stimulus salience or attention in the manuscript to respond to Reviewer 3 in the previous round of peer review. Specifically, painful stimuli may command greater attention than equally intense non-painful stimuli. Unfortunately, the present data cannot provide a direct empirical test of this hypothesis without salience ratings or arousal-related physiological signals. Our rating-matching analyses may be viewed as an *indirect* test of the influence of stimulus salience since intensity ratings have also been shown to highly correlate with stimulus salience (e.g., r = 0.84 in Liang et al., 2019 and r = 0.93 in Hadjis et al., 2025). If further direct tests confirm pain preferentiality of our findings, this phenomenon may be explained from a functional perspective. Intensity discriminability seems to play a functionally more important role in pain that in non-pain modalities. Intensity is a dominant part of sensory information animals extract from painful stimuli. In contrast, touch, audition, and vision encode plentiful information other than intensity, for example, texture and humidity for touch, timbre and pitch for audition, color and shape for vision. The informational value of pain intensity may cause the nervous system to selectively encode this information. This interpretation is, however, speculative and needs to be tested with carefully designed experiments in the future. We have discussed this speculative interpretation in the revised manuscript as follows (lines 690-702):

*If pain preferentiality is confirmed after directly controlling for attention, this phenomenon could reflect fundamentally different mechanisms underlying intensity discriminability of pain compared with non-pain modalities. While the present study only provides preliminary evidence for preferential encoding of pain discriminability, the informational value of pain intensity may explain the potential pain preferentiality. Intensity discriminability seems to play a functionally more important role in pain that in non-pain modalities. Intensity is a dominant part of sensory information which individuals extract from painful stimuli [21]. In contrast, touch, audition, and vision encode plentiful information other than intensity, for example, texture and humidity for touch, timbre and pitch for audition, color and shape for vision. The informational value of pain intensity may thus cause the nervous system to selectively encode this information. This interpretation is, however, speculative and needs to be tested with carefully designed experiments in the future.*

Notably, as argued by the other reviewer, the evidence for the pain-preferentiality in our study may still be not definitive and conclusive. Therefore, we have also toned down our description of relevant results. Specifically, we have changed the title to “Neural variability encodes individual differences in pain intensity discriminability”, and adopted the more nuanced expression of “*potentially* pain-preferential” throughout the revised manuscript, as suggested by the other reviewer.

2. Findings in patients and clinical relevance
The analyses in patients are an important addition, but the relatively small sample size (N = 27) limits the strength of these conclusions. Moreover, no direct statistical comparison has been performed between patients and healthy participants. I therefore encourage the authors to present these results more cautiously, particularly in the abstract and conclusion. It would also be helpful to explicitly report the number of patients in the abstract. Similarly, the potential clinical relevance could be moderated to reflect the preliminary nature of the patient data.

**Reply**: We agree that the sample size of the clinical dataset limits the robustness and clinical implication of our findings. They can only offer preliminary support to clinical utility. As suggested, we have explicitly mentioned the sample size of our clinical dataset in the abstract. We have also removed sentences suggesting unwarranted clinical implication of our findings in the abstract, results, and conclusion, as suggested by the other reviewer. In addition, we have clearly described the potential clinical relevance as only preliminary, and cautioned against unwarranted clinical implication of our findings as follows (lines 718-723):

*While consistent with these previous studies in principle, our findings should be interpreted with caution due to the limited sample size. They cannot provide immediate insights into chronic pain mechanisms or clinical utility. Rather, they only lend preliminary support to the potential clinical relevance of neural variability as an indicator of pain intensity discriminability. Future studies are needed to validate our findings in different types of chronic pain and in studies with larger clinical sample sizes.*

3. Definition and operationalization of discriminability
I remain somewhat puzzled by the definition of discriminability as differences in subjective rating scales. While I do not see a specific error in the reasoning, it remains unclear whether this measure truly captures the ability to discriminate as opposed to differences in internal scaling or rating behavior. The additional analyses based on signal detection theory (SDT) are a valuable step forward, though I am not in a position to fully evaluate their validity. I encourage the authors to explain the rationale for these analyses and their implications more clearly so that the broader readership can follow the argumentation. In addition, it would be helpful to explain the authors' definition of discriminability and its behavioral relevance more explicitly. For example, what does it mean in everyday life when one individual rates two stimuli as 3 and 5, while another individual rates the same stimuli as 3 and 7? Finally, the abstract should make it clear that the findings relate to differences in discriminability between participants (i.e., inter-individual differences) rather than discriminability between trials.
**Reply**: These analyses using rating differences and SDT-based AUC as metrics of discriminability rest on certain assumptions. Our claim that rating differences partly control for rating scaling is based on the assumption that the rating *r* of a stimulus *s* are the sum of the true perception *p* and a constant rating bias *b*, namely, $r_{s}=p_{s}+b$. If this assumption holds, then the rating bias cancels out in the rating differences, that is, $r_{s1}-r_{s2}={(p}_{s1}+b)-(p_{s2}+b)=p_{s1}-p_{s2}$. However, raw rating differences do not account for differences in rating distributions such as variance, and thus cannot fully control for rating scaling bias. SDT-based metrics can better control the distribution differences by normalizing rating differences using distributional parameters (e.g., variance in the case of $d'=\frac{r_{s1}-r_{s2}}{\sqrt{0.5*({Variance}_{s1}+{Variance}_{s2})}}$ and nonparametric control over distributions as in AUC). We therefore also employed SDT-based metrics in control analyses. Similar results between rating difference-based and AUC-based analyses suggest that distributional differences between ratings have no substantial effect. In other worlds, the simple “true perception + constant rating bias” assumption can be practically accepted. These points have been clarified in the revised manuscript as follows (lines 931-941):

*Taking the rating difference between two stimuli can partially control for the internal scaling in some sense, so long as one makes the assumption that the internal rating scale remains similar across different stimulus intensities for the same individual.* *Formally, assume that the rating r of a stimulus s are the sum of the true perception p and a constant rating bias b, namely,* $r_{s}=p_{s}+b$*, then the rating bias cancels out in the rating differences, that is,* $r_{s1}-r_{s2}={(p}_{s1}+b)-(p_{s2}+b)=p_{s1}-p_{s2}$*. However, raw rating differences do not fully account for the effect of rating distributions such as variance* *and therefore are still biased. SDT-based metrics can better control the distribution differences by normalizing rating differences using distributional parameters (e.g., variance in the case of* $d^{'}=\frac{r_{s1}-r_{s2}}{\sqrt{0.5*\left( {Variance}_{s1}+{Variance}_{s2} \right)}}$ *and nonparametric control over distributions as in AUC).*

To explain the definition of discriminability and its behavioral implications more clearly, we have further interpreted rating differences as slopes of psychophysical functions. Given fixed and medium physical intensities, mean rating differences are essentially the slope of individuals’ psychophysical function (Fig R1; also added as S Fig 1 in the revised manuscript). A person with higher discriminability will be better at distinguishing two stimuli of similar yet different physical intensities (x1 and x2). As a result, their psychophysical function will have steeper slopes and the mean ratings will be larger (i.e., y3 – y1 > y2 – y1). In the example in which one person gives ratings of 3 and 5 and another person gives 3 and 7, the first person has a flatter slope than the second. Conceptually, this means the first person is worse at distinguishing the two stimuli. These points have been introduced in the revised manuscript as follows (lines 131-138):

*Here, individual differences in pain intensity discriminability were operationalized as differences in mean subjective ratings between low- and high-intensity stimuli. For example, if one person rates two painful stimuli as 3 and 5 on average on a 0~10 rating scale, their pain intensity discriminability is quantified as 5 – 3 = 2. Essentially, this quantity is the slope of the psychophysical function given two fixed and medium physical intensities. Individuals who have better intensity discriminability will have a steeper psychophysical function and thus larger rating differences (S Fig 1).*

We have also highlighted that by “pain intensity discriminability” we mean “individual differences in pain intensity discriminability” in the title, abstract, and main text.

**Figure R1. Rating differences as slopes of psychophysical functions.** Given two fixed and medium physical intensities x1 and x2, the rating differences y3 – y1 and y2 – y1 can be viewed as the slope of psychophysical functions. Individuals with higher discriminability have a larger slope (i.e., y3 – y1 > y2 – y1).

**Reviewer #2:**

The research 'Neural variability reliably and preferentially encodes pain intensity discriminability' comprises a deep and considered analysis of measures of neural variation in relation to pain discriminability (as quantified by objective differences in stimulation levels) across multiple datasets. The manuscript is well written and the rationale for the research is clear. The authors have made considered responses to previous reviews which, generally, explain their decisions well and satisfy concerns. I note a few minor points, particularly regarding the strength of interpretation in key aspects including title and abstract, which warrant further consideration before the manuscript is suitable for publication.
**Reply**: We appreciate the reviewer’s positive evaluation of our manuscript. We have revised our manuscript to address the remaining minor points and provided detailed point-by-point replies below.

I largely agree with R1 (Point 2) and R2 (point 2) that the links made between chronic pain and the outcomes of this research are preliminary and, as such, they should be described and discussed carefully. The authors have made some effort reduce and improve this in their latest version and have included more up-to-date studies. However, some aspects still press this point that neural variability in response to experimental pain could offer immediate insights into chronic pain mechanisms or clinical utility. Specifically, I think point 3 in the abstract is overstatement and I recommend that this should be fully removed. The present research includes just one small PHN group and I would agree with the authors own interpretation employed later in the manuscript proper were they state that this offers only preliminary support towards links to chronic pain or clinical utility. It is a discussion point but not something worthy of claiming in abstract.
**Reply**: We agree that our findings can only offer preliminary support to clinical utility. As suggested, we have removed sentences suggesting unwarranted clinical implication of our findings in the abstract, results, and conclusion. We have also explicitly mentioned the sample size of our clinical dataset in the abstract, as suggested by the other reviewer. In addition, we have clearly described the potential clinical relevance as only preliminary, and cautioned against unwarranted clinical implication of our findings as follows (lines 718-723):

*While consistent with these previous studies in principle, our findings should be interpreted with caution due to the limited sample size. They cannot provide immediate insights into chronic pain mechanisms or clinical utility. Rather, they only lend preliminary support to the potential clinical relevance of neural variability as an indicator of pain intensity discriminability. Future studies are needed to validate our findings in different types of chronic pain and in studies with larger clinical sample sizes.*

I also feel that there is still some overstatement of the findings in relation to pain selectivity. R3, myself (and I think many readers) will still have concerns about the difficulty of matching salience across modalities. I would argue that the authors finding that discriminability-variability links in pain modality could exhibit a preferential status can be described as preliminary or requiring further research. I believe readers will still hope to see future independent replication and investigations with specific experimental designs to directly consider stimuli salience. The author response to R3 about this same point 'in the interpretation of our findings, we have replaced the term "pain selectivity" with the more neutral "pain preferential"' is not really sufficient in my estimation. One direct change wI recommend is to to cut 'preferential' from the title. The link between neural variability and pain discriminability is enough for title and I do not believe that this work is yet sufficient to claim that this relationship is definitively preferential as the title suggests. Likewise, in the abstract where they state 'and preferential indicator' this should be more nuanced and changes to 'potentially preferential indicator' I much prefer the authors language in results: 'the results above offer preliminary evidence that neural variability preferentially encodes pain intensity discriminability.' This is how I think it could be pitched throughout the manuscript. On line 331 I suggest changing 'probably' to 'possibly' due to the need for further research with regards to salience and other factors. These minor changes, along with above changes to title and abstract, will better align the data and claims in the manuscript for the reader.

**Reply**: We agree that we have not provided definitive evidence for the pain-preferentiality. As suggested, we have toned down our description of relevant results. The title has been changed to “Neural variability encodes individual differences in pain intensity discriminability”, “probably” in line 331 in the original manuscript has been rephrased as “possibly” (line 431), and we have adopted the more nuanced expression of “potentially pain-preferential” throughout the revised manuscript.
